# Syntheses of Cannabinoid Metabolites: Ajulemic Acid and HU-210

**DOI:** 10.3390/molecules29020526

**Published:** 2024-01-21

**Authors:** Wenbin Shao, Pingyong Liao, Xiaoyan Zhang, Binbin Fan, Ruijia Chen, Xilong Chen, Xuejun Zhao, Wenbin Liu

**Affiliations:** 1 Shanghai Key Laboratory of Crime Scene Evidence, Shanghai Research Institute of Criminal Science and Technology, Shanghai 200072, China; shaowenbin@yuansi.com.cn (W.S.); liaopingyong@yuansi.com.cn (P.L.); chenruijia@yuansi.com.cn (R.C.); chenxilong@yuansi.com.cn (X.C.); 2 Shanghai Yuansi Standard Science and Technology Co., Ltd., Shanghai 200072, China; 3 State Key Laboratory of Bio-Fibers and Eco-Textiles, Institute of Marine Biobased Materials, Collage of Materials Science and Engineering, Qingdao University, Qingdao 266071, China; zhangxiaoyan@qdu.edu.cn (X.Z.); fanbb1991@tju.edu.cn (B.F.)

**Keywords:** phytocannabinoids, metabolites, ajulemic acid, Riley oxidation

## Abstract

Cannabinoid metabolites have been reported to be more potent than their parent compounds. Among them, ajulemic acid (AJA) is a side-chain analog of Δ^9^-THC-11-oic acid, which would be a good template structure for the discovery of more potent analogues. Herein, we optimized the key allylic oxidation step to introduce the C-11 hydroxy group with a high yield. A series of compounds was prepared with this condition applied including HU-210, 11-nor-Δ^8^-tetrahydrocannabinol (THC)-carboxylic acid and Δ^9^-THC-carboxylic acid.

## 1. Introduction

**Figure 1:**
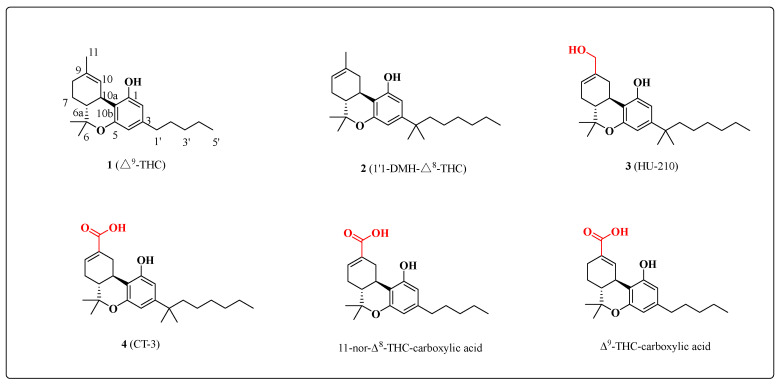
General structural information for cannabinoid metabolites with oxygen at C-11 site.

Phytocannabinoids and their synthetic analogues, exemplified by molecules such as Δ^9^-THC, **1** (Figure 1), are prime candidates for pharmaceutical innovation and are known to possess potent analgesic and anti-inflammatory properties. Compound **1** was first identified in 1964 by Gaoni and Mechoulam as the principle bioactive component of marijuana (hashish), which has been used for centuries as both a therapeutic and recreational drug. More generally, cannabinoid-based chemical probes and leads are essential for the continued exploration of the endocannabinoid system [1]. Prior to the 1980s, cannabinoids were hypothesized to produce their effects through nonspecific interactions with cell membranes. The absolute stereochemistry of **1** was established in 1967 [2], and more than two decades passed before it was identified as a modulator of the cannabinoid receptor [3].

The main metabolic pathways involve hydroxylations or oxygenations. It is well-known that substituents at the C-1, C-3 and C-9 positions play critical roles in efficient binding to the cannabinoid receptors [4]. SAR studies of the C-3 side chain demonstrated that a seven-carbon homologue was optimal for activity and that branched alkyl groups also led to improved binding affinity [5]. For example, the dimethylheptyl analogue **3**, which is an oxygenation product of **2**, exhibits an approximately 50-fold improvement in activity relative to **1** [6]. The relative importance of the C-1 hydroxyl differs between the two cannabinoid subtypes. Synthetic cannabinoid **3** [7] possesses two hydrogen donors and exhibits significantly enhanced affinity for both the CB2 and the CB1 receptors, producing many of the same pharmacological effects as **1** [6].

Compound **4** is a synthetic analog of Δ^9^-THC-11-oic acid, the major metabolite of the psychoactive component of marijuana, Δ^9^-THC. Δ^9^-THC-11-oic acid [8] has no psychotropic activity and is present in the tissues of the millions of recreational cannabis users long after the mood altering effects are gone [9]. Its analgesic properties suggest that it would be a good template structure for the discovery of more potent analogs. AJA is such an analog, and is a ‘first-in-class’ chemical entity designed to have increased anti-inflammatory properties and reduced psychotropic activity compared to its THC parent [10]. This compound was found to be well tolerated in a phase I clinical trial, and subsequently, a phase II study was completed wherein this molecule demonstrated efficacy in reducing chronic neuropathic pain without any major adverse effects [11].

**Figure 2:**
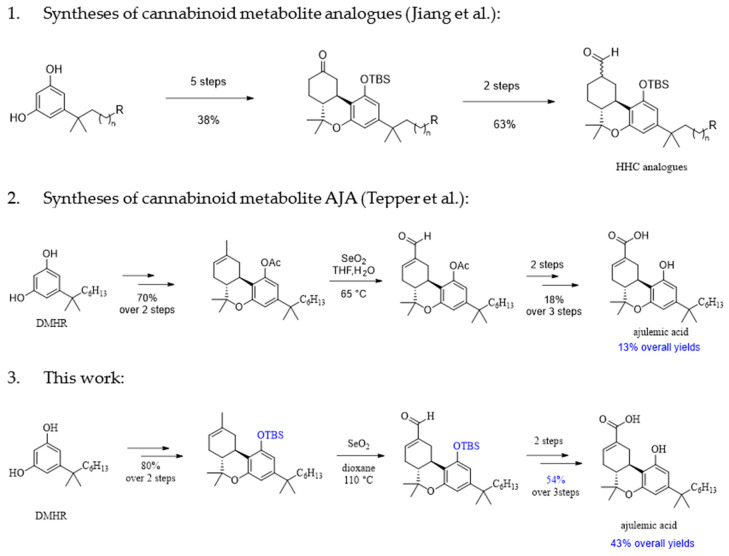
Overview of the syntheses of the C-11 oxygenation intermediates. 1. Jiang et al. [12]; 2. Tepper et al. [13].

There are few reports on the syntheses of cannabinoid metabolites, which vary in length from five to seven steps (Figure 2). Jiang et al. reported a synthetic route for the tricyclic hexahydrocannabinol (HHC) analogues with seven steps and 24% overall yields [12]. Tepper et al. developed a synthetic route to achieve the AJA within five steps but only in 13% overall yields [13]. Considering the rapid change in the illicit drug market, a concise synthetic route (Figure 2) where the combination of a simple replacement of the phenol protecting group and the optimization of the Riley oxidation condition give a better yield (43% overall yield). Therefore, a synthetic route for the synthesis of a key intermediate, which subsequently, can be used to synthesize the cannabinoid metabolites, was developed. The synthesis method for the intermediate is carried out on a multigram scale.

## 2. Results and Discussion

**Scheme 1:**
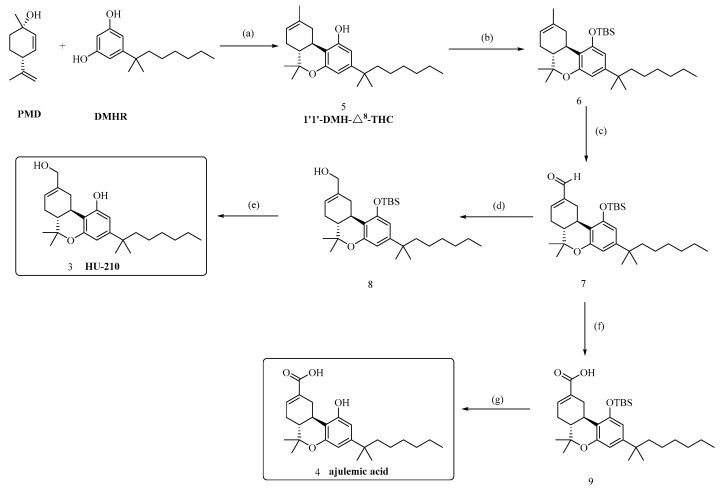
Synthesis of AJA and HU-210. Reagents and conditions: (a) 1. *p*-TSA, toluene; 80 °C for 1 h; yield of 82%. (b) TBSCl, imidazole and *N,N*-dimethylformamide (DMF); room temperature for overnight duration; yield of 97%; (c) SeO_2_, dioxane; 110 °C for 1 h; yield of 65%. (d) NaBH_4_ and MeOH; 0 °C for 50 min; yield of 89%. (e) TBAF and THF; room temperature for 2 h; yield of 93%; (f) NaClO_2_, NaH_2_PO_4_, 2-methyl-2-butene, *t*-BuOH and H_2_O (4:1); room temperature for 1 h; yield of 90%; (g) TBAF and THF; room temperature for 2 h; yield of 92%.

The route to the synthesis of AJA by Tepper was optimized by changing the SeO_2_-mediated condition to improve the yield of the key allylic oxidation step. Our synthesis started with the preparation of the tricyclic intermediate (**5**) [14], as shown in Scheme 1. The commercially available starting materials *p*-menthadienol (PMD) and 1,1-dimethylheptyl resorcinol (DMHR) were used under acid conditions to promote cyclization to obtain the tricyclic skeleton of the cannabinoid metabolites in 82% yield. Thus, the further protection of the phenol group with a TBS group afforded the key tricyclic intermediate (**6**) in 97% yield. To install the C11 hydroxyl group, (**6**) was subjected to a SeO_2_-mediated allylic oxidation, and product (**7**) bearing an aldehyde group at C11 was obtained in 65% yield. Then, the reduction of the new generated aldehyde group with NaBH_4_ afforded the hydroxy group in 89% yield. The resultant (**8**) was then treated with 1 M tetra-N-butylammonium fluoride (TBAF) in tetrahydrofuran (THF) to give product HU-210 in 93% yield. We then turned our attention to (**7**) for the total synthesis of AJA. To this end, (**7**) was first converted to acid (**9**) with treatment by Pinnick oxidation in 90% yield; further treatment of (**9**) with TBAF in THF gave product AJA in 92% yield.

During the initial attempt, the acetyl protecting group of the tricyclic intermediate phenol **10** led to a low yield at the allylic oxidation step. We tried a variety of conditions including SeO_2_/THF/H_2_O [13], SeO_2_/AcOH/DCM [15], SeO_2_/*t*BuOOH/salicylic acid/DCM [16], SeO_2_/*t*BuOOH/DCM [17] and SeO_2_/dioxane. The results showed that there was a byproduct (**12**), which is a regio-selected isomer. There was also another byproduct (**13**), which is an aromatic product, and is shown in Table 1. Fortunately, the SeO_2_-mediated Riley oxidation in dioxane at 110 °C gave a moderate yield. When the acetyl group was replaced with the silyl-ether-protected group, the yield of the allylic oxidation was improved markedly due to the significant decrease in by-products.

**Scheme 2:**
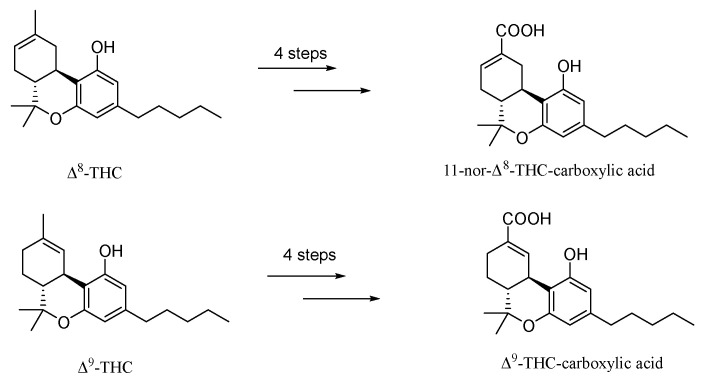
Syntheses of 11-nor-Δ^8^-THC-9-carboxylic acid and Δ^9^-THC-carboxylic acid with the same operations.

The optimized conditions were then applied for the syntheses of 11-nor-Δ^8^-THC-9-carboxylic acid and Δ^9^-THC- carboxylic acid (Figure 1). To this end, this method was used to successfully obtain the metabolites through a four-step strategy from Δ^8^-THC and Δ^9^-THC (Scheme 2).

## 3. Materials and Methods

### 3.1. General Information

All solvents and reagents used in this study were purchased from commercial sources and used without further purification. Thin-layer chromatography (TLC) was performed using SIL G/UV 254 silica–glass plates. Flash chromatography was carried out using Silica Gel 60 (200–400 mesh), and solvent systems defined in the experimental procedure were utilized for each synthesized molecule. NMR spectra were obtained using a JEOL JNM-ECZ600R 600 MHz spectrometer at room temperature. NMR spectra were calibrated using residual undeuterated solvent as an internal reference (CDCl_3_: ^1^H-NMR = 7.26 ppm, ^13^C-NMR = 77.16 ppm; Acetone-*d*_6_: ^1^H-NMR = 2.05 ppm, ^13^C-NMR = 206.3 ppm; DMSO-*d*_6_: ^1^H-NMR = 2.50 ppm, ^13^C-NMR = 39.52 ppm; CD_2_Cl_2_: ^1^H-NMR = 5.32 ppm, ^13^C-NMR = 54.0 ppm; MeOD-*d*_4_: ^1^H-NMR = 3.31 ppm, ^13^C-NMR = 49.0 ppm; the following abbreviations were used to explain the multiplicities: s = singlet, d = doublet, t = triplet, q = quartet, m = multiplet, br = broad. High-resolution mass spectroscopy (HRMS) was carried out on a Vion IMS TOF-Q mass spectrometer.

### 3.2. Experimental Section

#### 3.2.1. Synthesis of Compound (**5**)

DMHR (5 g, 21.18 mmol, 1 equiv), *p*-toluenesulfonic acid (0.728 g, 3.83 mmol, 0.2 equiv) and toluene (150 mL) were added to a 500 mL, three-neck round-bottom flask. PMD (3.54 g, 23.29 mmol, 1.1 equiv) was added to this over 1 h, followed by a toluene (8 mL) rinse, while maintaining the batch temperature at 15–30 °C. The batch was heated to 70–80 °C under partial vacuum, and a Dean–Stark trap filled with toluene was used to remove water azeotropically. The reaction was quenched by the addition of a saturated solution of NH_4_Cl (15 mL). The mixture was extracted with EtOAc (3 × 50 mL). The combined organic layers were washed with brine (20 mL), and dried with Na_2_SO_4_. The solvent was removed by vacuum, and the residue was purified by flash chromatography on silica gel (hexane/ethyl acetate = 50/1) to give **5** (6.45 g, 82% yield) as a yellow oil. R_f_ = 0.7 (silica gel, EtOAc/hexanes= 1/10). ^1^H-NMR (600 MHz, chloroform-*d*) δ 6.40 (d, *J* = 1.8 Hz, 1H), 6.23 (d, *J* = 1.8 Hz, 1H), 5.44 (dt, *J* = 5.0, 1.4 Hz, 1H), 3.20 (dt, *J* = 17.2, 3.0 Hz, 1H), 2.75–2.66 (m, 1H), 2.14 (d, *J* = 4.1 Hz, 1H), 1.95–1.77 (m, 3H), 1.71 (q, *J* = 1.3 Hz, 3H), 1.50 (ddd, *J* = 11.3, 6.1, 2.6 Hz, 2H), 1.40 (s, 3H), 1.26–1.16 (m, 13H), 1.12 (s, 3H), 1.07 (td, *J* = 8.9, 8.4, 4.1 Hz, 2H), 0.85 (t, *J* = 7.1 Hz, 3H). ^13^C-NMR (151 MHz, chloroform-*d*) δ 154.65, 154.55, 150.17, 134.90, 119.47, 110.29, 108.17, 105.54, 44.98, 44.61, 37.45, 36.10, 31.93, 31.63, 30.17, 28.88, 28.81, 28.01, 27.75, 24.76, 23.65, 22.82, 18.66, 14.24. IR (film, cm^−1^): 3384, 2958, 2927, 2856, 1622, 1413, 1034, 965, 838. HRMS (ESI) calcd for C_25_H_38_O_2_ [M+H]^+^: *m/z* 371.2945, found: 371.2944.

#### 3.2.2. Synthesis of Compound (**6**)

Dry imidazole (4.75 g, 69.77 mmol, 4.46 equiv) and TBSCl (7.07 g, 46.9 mmol, 3.0 equiv) were added to a solution of **5** (5.79 g, 15.65 mmol, 1.0 equiv) in dry DMF (100 mL) at room temperature and the resultant mixture was stirred at the same temperature for 18 h. The mixture was quenched by the addition of a saturated solution of NH_4_Cl (10 mL), and water (200 mL) was added to the mixture. The mixture was extracted with ethyl acetate (3 × 50 mL). The combined organic layers were washed with brine (25 mL), and dried over Na_2_SO_4_. The solvent of the organic phase was concentrated in vacuo, and the residue was purified by flash column chromatography (hexane/EtOAc: 50/1) to give **6** (7.34 g, 97% yield) as a colorless oil. R_f_ = 0.7 (hexane/EtOAc: 20/1). ^1^H-NMR (600 MHz, chloroform-*d*) δ 6.41 (d, *J* = 1.9 Hz, 1H), 6.35 (d, *J* = 1.9 Hz, 1H), 5.45–5.35 (m, 1H), 3.30–3.20 (m, 1H), 2.61–2.53 (m, 1H), 2.22–2.09 (m, 1H), 1.84–1.77 (m, 3H), 1.69 (d, *J* = 2.0 Hz, 3H), 1.50 (ddd, *J* = 10.3, 5.5, 1.2 Hz, 2H), 1.38 (s, 3H), 1.21 (d, *J* = 6.4 Hz, 8H), 1.19 (dd, *J* = 7.1, 3.5 Hz, 4H), 1.10 (s, 3H), 1.07–1.03 (m, 2H), 1.01 (s, 9H), 0.84 (t, *J* = 7.1 Hz, 3H), 0.26 (s, 3H), 0.13 (s, 3H). ^13^C-NMR (151 MHz, chloroform-*d*) δ 154.71, 154.30, 149.40, 135.15, 119.36, 114.19, 109.83, 108.66, 45.34, 44.72, 37.44, 36.15, 32.24, 31.97, 31.74, 30.18, 29.06, 28.81, 28.19, 27.64, 26.13, 24.83, 23.54, 22.80, 18.47, 14.27, 14.23, −3.41, −4.24. HRMS (ESI) calcd for C_31_H_52_O_2_Si [M+H]^+^: *m/z* 485.3809, found: 485.3818.

#### 3.2.3. Synthesis of Compound (**7**)

SeO_2_ (4.6 g, 41.46 mmol, 3.5 equiv) was added to a stirred solution of **6** (5.74 g, 11.84 mmol, 1.0 equiv) in dry dioxane (200 mL) at room temperature, and the resultant mixture was shielded from light and stirred at 110 °C for 1 h. The mixture was quenched by filtration through a celite pad, which was washed with DCM (50 mL). The combined filtrate was washed with saturated aq Na_2_S_2_O_8_ (3 × 20 mL), and the water phase was extracted with DCM (3 × 50 mL). The combined organic layers were washed with brine (50 mL) and dried over Na_2_SO_4_. The organic phase was concentrated in vacuo, and the residue was purified by flash column chromatography (hexane/EtOAc: 10/1 to 4/1) to give **7** (3.82 g, 65% yield) as a yellowish solid, R_f_ = 0.3 (hexane/EtOAc: 10/1). ^1^H-NMR (600 MHz, chloroform-*d*) δ 9.50 (s, 1H), 6.83–6.77 (m, 1H), 6.41 (d, *J* = 1.8 Hz, 1H), 6.37 (d, *J* = 1.8 Hz, 1H), 3.84 (ddd, *J* = 17.7, 4.3, 2.0 Hz, 1H), 2.60–2.48 (m, 2H), 2.19–2.10 (m, 1H), 1.90 (td, *J* = 11.6, 4.7 Hz, 1H), 1.80 (q, *J* = 2.1 Hz, 1H), 1.52–1.48 (m, 2H), 1.43 (s, 3H), 1.20 (dd, *J* = 15.9, 4.1 Hz, 13H), 1.13 (s, 3H), 1.04 (t, *J* = 4.0 Hz, 1H), 0.97 (s, 9H), 0.84 (t, *J* = 7.0 Hz, 3H), 0.28 (s, 3H), 0.13 (s, 3H). ^13^C-NMR (151 MHz, chloroform-*d*) δ 193.42, 154.90, 154.06, 149.91, 148.69, 142.57, 112.86, 109.85, 108.43, 45.33, 44.70, 37.50, 31.95, 31.45, 30.16, 29.45, 28.98, 28.85, 27.69, 27.61, 26.08, 24.81, 22.79, 18.46, 18.31, 14.23, −3.43, −4.12. HRMS (ESI) calcd for C_31_H_50_O_3_Si [M+H]^+^: *m/z* 499.3602, found: 499.3615.

#### 3.2.4. Synthesis of Compound (**8**)

NaBH_4_ (256 mg, 6.74 mmol, 1.2 equiv) was added to a solution of **7** (2.80 g, 5.62 mmol, 1.0 equiv) in MeOH (30 mL) at 0 °C in one portion, and the resultant mixture was stirred at 0 °C for 50 min. The reaction was quenched by the addition of an aqueous solution of NH_4_Cl (10 mL), the MeOH in the resultant mixture was removed under vacuum, and the resultant residue was extracted with ethyl acetate (3 × 10 mL). The combined organic extracts were washed with brine (10 mL) and dried over Na_2_SO_4_. The solvent of the extract was concentrated under vacuum, and the residue was purified by flash column chromatography (hexane/EtOAc: 10/1 to 4/1) to give **8** (2.5 g, 89% yield) as a yellowish solid, R_f_ = 0.5 (hexane/EtOAc: 5/1). ^1^H-NMR (600 MHz, chloroform-*d*) δ 6.42 (d, *J* = 1.9 Hz, 1H), 6.35 (d, *J* = 1.9 Hz, 1H), 5.76–5.71 (m, 1H), 4.08–3.99 (m, 2H), 3.41–3.32 (m, 1H), 2.59 (td, *J* = 11.0, 4.5 Hz, 1H), 2.27–2.15 (m, 1H), 1.95–1.77 (m, 3H), 1.52–1.47 (m, 2H), 1.39 (s, 3H), 1.25–1.19 (m, 9H), 1.18 (q, *J* = 3.5 Hz, 3H), 1.11 (s, 3H), 1.07–1.02 (m, 2H), 0.99 (s, 9H), 0.84 (t, *J* = 7.1 Hz, 3H), 0.25 (s, 3H), 0.13 (s, 3H). ^13^C-NMR (151 MHz, chloroform-*d*) δ 154.75, 154.23, 149.57, 138.57, 120.42, 113.78, 109.85, 108.64, 76.42, 66.91, 45.55, 44.70, 37.45, 32.00, 31.95, 30.16, 29.03, 28.80, 27.85, 27.62, 26.13, 24.82, 22.79, 18.45, 18.40, 14.23, −3.43, −4.17. HRMS (ESI) calcd for C_31_H_52_O_3_Si [M+H]^+^: *m/z* 501.3759, found: 501.3744.

#### 3.2.5. Synthesis of Compound (**10**)

Ac_2_O (5.11 mL, 6.74 mmol, 4 equiv) was added to a solution of **5** (5.6 g, 13.5 mmol, 1.0 equiv) in pyridine (100 mL) at 0 °C in one portion, and the resultant mixture was then stirred at room temperature for 4 h. The mixture was then diluted with water (100 mL) and the resultant residue was extracted with ethyl acetate (3 × 30 mL). The combined organic extracts were washed with H_2_O (3 × 10 mL) and brine (10 mL), and dried over Na_2_SO_4_. The solvents of the extracts were concentrated under vacuum, and the residue was purified by flash column chromatography (hexane/EtOAc: 10/1) to give **10** (5.93 g, 95% yield) as a yellowish solid, R_f_ = 0.7 (hexane/EtOAc: 10/1). ^1^H-NMR (600 MHz, chloroform-*d*) δ 6.69 (d, *J* = 2.2 Hz, 1H), 6.52 (d, *J* = 2.2 Hz, 1H), 5.48–5.40 (m, 1H), 2.77–2.69 (m, 1H), 2.61 (td, *J* = 11.0, 5.0 Hz, 1H), 2.30 (s, 3H), 2.17–2.11 (m, 1H), 1.97–1.89 (m, 1H), 1.83–1.77 (m, 2H), 1.70 (s, 3H), 1.52 (dd, *J* = 8.0, 4.5 Hz, 2H), 1.39 (s, 3H), 1.21 (dd, *J* = 26.3, 3.2 Hz, 12H), 1.12 (s, 3H), 1.08 (ddd, *J* = 11.0, 5.2, 2.7 Hz, 2H), 0.85 (t, *J* = 7.0 Hz, 3H). ^13^C-NMR (151 MHz, chloroform-*d*) δ 169.06, 154.31, 150.27, 149.73, 134.01, 119.89, 115.83, 113.19, 112.27, 44.76, 44.57, 37.59, 36.20, 31.88, 31.86, 30.11, 28.75, 28.67, 27.85, 27.60, 24.68, 23.70, 22.80, 21.44, 18.68, 14.23. HRMS (ESI) calcd for C_27_H_40_O_3_ [M+H]^+^: *m/z* 413.6215, found: 413.6214.

#### 3.2.6. Synthesis of Compound (**11**)

Compound **10** (1.23 g, 2.98 mmol, 1 equiv), selenium dioxide (378 mg, 3.41 mmol, 1.25 equiv), tetrahydrofuran (12.2 mL, 4.3 equiv) and water (0.57 mL, 0.2 equiv) were added to a 100 mL three-neck round bottom flask. The reactor was heated to 55–65 °C for 23.5 h. The mixture was quenched by filtration through a celite pad, which was washed with DCM (20 mL). The combined filtrate was washed with saturated aq Na_2_S_2_O_8_ (3 × 10 mL), and the water phase was extracted with DCM (3 × 12 mL). The combined organic layers were washed with brine (15 mL) and dried over Na_2_SO_4_. The organic phase was concentrated in vacuo, and the residue was purified by flash column chromatography (hexane/EtOAc: 10/1 to 4/1) to give **11** (0.32 g, 25% yield) as a yellowish solid, R_f_ = 0.3 (hexane/EtOAc: 10/1) and also to give two byproducts **12** and **13**.

Compound **11**, ^1^H-NMR (600 MHz, chloroform-*d*) δ 9.50 (s, 1H), 6.90–6.81 (m, 1H), 6.70 (d, *J* = 1.7 Hz, 1H), 6.56 (d, *J* = 2.0 Hz, 1H), 3.43–3.34 (m, 1H), 2.57–2.54 (m, 1H), 2.30 (s, 3H), 2.16–2.10 (m, 1H), 1.91 (td, *J* = 11.5, 4.5 Hz, 2H), 1.53–1.49 (m, 2H), 1.44 (s, 3H), 1.23 (d, *J* = 2.9 Hz, 8H), 1.19 (t, *J* = 4.0 Hz, 5H), 1.16 (s, 3H), 1.07 (dq, *J* = 9.1, 3.7, 3.3 Hz, 2H), 0.84 (d, *J* = 7.3 Hz, 3H). ^13^C-NMR (151 MHz, chloroform-*d*) δ 193.43, 169.28, 154.03, 150.83, 149.79, 148.81, 141.65, 114.59, 113.21, 112.67, 44.72, 44.54, 37.66, 31.87, 31.07, 30.09, 29.12, 28.75, 28.63, 27.55, 27.11, 24.67, 22.80, 21.46, 18.55, 14.26, 14.22. HRMS (ESI) calcd for C_27_H_38_O_4_ [M+H]^+^: *m/z* 427.2843, found: 427.2838.

Compound **12**, ^1^H-NMR (600 MHz, chloroform-*d*) δ 6.99 (s, 1H), 6.43 (d, *J* = 1.9 Hz, 1H), 6.38 (d, *J* = 2.2 Hz, 1H), 5.68 (dt, *J* = 5.2, 1.7 Hz, 1H), 5.64–5.59 (m, 1H), 3.04–2.96 (m, 1H), 2.24 (s, 3H), 2.23–2.17 (m, 1H), 2.10 (td, *J* = 11.3, 3.8 Hz, 1H), 1.97–1.90 (m, 1H), 1.52–1.48 (m, 2H), 1.40 (s, 3H), 1.23 (s, 3H), 1.20 (s, 12H), 1.08–1.04 (m, 2H), 1.02 (s, 3H), 0.84 (d, *J* = 7.2 Hz, 3H). ^13^C-NMR (151 MHz, chloroform-*d*) δ 169.69, 155.11, 151.56, 134.68, 125.64, 108.70, 107.42, 107.09, 79.86, 75.64, 46.69, 44.43, 37.49, 37.35, 31.87, 30.10, 28.93, 28.80, 28.72, 27.55, 24.67, 22.77, 21.19, 19.19, 18.34, 14.22. HRMS (ESI) calcd for C_27_H_40_O_4_ [M+H]^+^: *m/z* 429.2999, found: 429.3000.

Compound **14**, ^1^H-NMR (600 MHz, chloroform-*d*) δ 6.47 (d, *J* = 2.2 Hz, 1H), 6.33 (d, *J* = 2.2 Hz, 1H), 5.73 (d, *J* = 6.1 Hz, 1H), 4.39–4.32 (m, 1H), 2.80 (dd, *J* = 11.8, 8.5 Hz, 1H), 2.22–2.13 (m, 1H), 1.97–1.89 (m, 1H), 1.89–1.82 (m, 1H), 1.79 (d, *J* = 2.1 Hz, 3H), 1.51 (ddd, *J* = 11.3, 6.0, 2.4 Hz, 2H), 1.38 (s, 3H), 1.27–1.16 (m, 13H), 1.09 (s, 3H), 1.08–1.05 (m, 2H), 0.85 (t, *J* = 7.0 Hz, 3H). ^13^C-NMR (151 MHz, chloroform-*d*) δ 155.92, 154.69, 151.21, 134.70, 125.84, 108.23, 107.83, 107.60, 77.51, 77.37, 75.21, 44.89, 44.49, 40.95, 37.39, 31.95, 30.18, 28.78, 27.96, 27.87, 24.77, 22.83, 19.28, 18.64, 14.25. HRMS (ESI) calcd for C_25_H_38_O_3_ [M+H]^+^: *m/z* 387.2893, found: 387.2899.

Compound **15**, ^1^H-NMR (600 MHz, chloroform-*d*) δ 9.94 (s, 1H), 8.57 (d, *J* = 1.4 Hz, 1H), 7.23 (d, *J* = 1.5 Hz, 1H), 6.56 (d, *J* = 1.7 Hz, 1H), 6.43 (d, *J* = 1.8 Hz, 1H), 5.97 (d, *J* = 5.0 Hz, 1H), 5.61 (d, *J* = 3.1 Hz, 1H), 1.76 (s, 6H), 1.55 (dd, *J* = 8.0, 4.3 Hz, 2H), 1.26 (s, 6H), 1.20 (q, *J* = 4.9, 4.0 Hz, 6H), 1.09–1.06 (m, 2H), 0.84 (s, 3H). HRMS (ESI) calcd for C_25_H_32_O_4_ [M+H]^+^: *m/z* 381.2424, found: 381.2428.

#### 3.2.7. Synthesis of Compound (**3**)

TBAF (1 M in THF, 4.84 mL, 1.1 equiv) was added to a solution of **8** (2.20 g, 4.40 mmol, 1.0 equiv) in THF (60 mL), and the resultant mixture was stirred at room temperature for 2 h. The reaction was quenched with saturated aq NH_4_Cl (10 mL), and the resultant mixture was extracted with Et_2_O (3 × 10 mL). The organic layers were sequentially washed with water (5 mL) and then with brine (5 mL), and dried over anhydrous MgSO_4_. The solvent of the extract was removed under vacuum, the residue was subjected to column chromatography on silica gel (hexane/EtOAc: 2/1) to afford **3** (1.57 mg, 93%) as a white solid. R_f_ = 0.4 (hexane/EtOAc: 2/1). ^1^H-NMR (600 MHz, chloroform-*d*) δ 6.39 (d, *J* = 1.8 Hz, 1H), 6.24 (d, *J* = 1.9 Hz, 1H), 5.75 (dd, *J* = 4.0, 2.4 Hz, 1H), 4.07 (q, *J* = 12.7 Hz, 2H), 3.43 (dd, *J* = 15.9, 4.5 Hz, 1H), 2.71 (d, *J* = 4.6 Hz, 1H), 2.22 (s, 1H), 1.92–1.80 (m, 3H), 1.49 (ddd, *J* = 11.3, 6.1, 2.7 Hz, 2H), 1.40 (s, 3H), 1.28–1.14 (m, 13H), 1.11 (s, 3H), 1.09–1.02 (m, 2H), 0.85 (t, *J* = 7.1 Hz, 3H). ^13^C-NMR (151 MHz, chloroform-*d*) δ 154.78, 154.50, 150.30, 138.33, 121.98, 110.03, 107.94, 105.75, 67.22, 45.12, 44.61, 37.42, 31.94, 31.50, 31.44, 30.19, 28.86, 28.79, 27.80, 27.71, 24.76, 22.83, 18.53, 14.24. IR (film, cm^−1^): 3413, 3223, 2924, 1624, 1580, 1415, 1186, 991, 842. HRMS (ESI) calcd for C_25_H_38_O_3_ [M+H]^+^: *m/z* 387.2894, found: 387.2891.

#### 3.2.8. Synthesis of Compound (**9**)

NaClO_2_ (1.95 g, 648 mmol, 4.0 equiv) was added to a mixture of aldehyde **7** (2.69 g, 5.39 mmol, 1.0 equiv), NaH_2_PO_4_·2H_2_O (2.59 g, 648 mmol, 4.0 equiv) and 2-methyl-2-butene (3.79 g, 53.9 mmol, 10.0 equiv) in t-BuOH (100 mL) and H_2_O (25 mL) at 0 °C. After being stirred for 1 h at that temperature, the mixture was extracted with EtOAc (3 × 40 mL) and H_2_O (15 mL). The organic layer was separated, dried over Na_2_SO_4_, and concentrated to give the residue, which was purified by column chromatography on silica gel (hexane/EtOAc: 20/1) to afford **9** (2.50 g, 90%) as a yellowish oil. R_f_ = 0.4 (hexane/EtOAc: 10/1). ^1^H-NMR (600 MHz, chloroform-*d*) δ 7.19–7.14 (m, 1H), 6.44 (d, *J* = 1.8 Hz, 1H), 6.40 (d, *J* = 1.9 Hz, 1H), 3.90 (ddd, *J* = 17.9, 4.4, 2.1 Hz, 1H), 2.57 (td, *J* = 11.2, 4.3 Hz, 1H), 2.50–2.40 (m, 1H), 2.04 (ddt, *J* = 16.4, 11.9, 2.4 Hz, 1H), 1.97–1.90 (m, 1H), 1.86 (td, *J* = 11.7, 4.6 Hz, 1H), 1.54–1.51 (m, 2H), 1.42 (s, 3H), 1.22 (dd, *J* = 13.7, 5.9 Hz, 13H), 1.13 (s, 3H), 1.10–1.04 (m, 2H), 1.01 (s, 9H), 0.86 (t, *J* = 7.0 Hz, 3H), 0.30 (s, 3H), 0.16 (s, 3H). ^13^C-NMR (151 MHz, chloroform-*d*) δ 172.77, 154.84, 154.13, 149.74, 140.24, 130.98, 112.99, 109.81, 108.50, 75.98, 44.69, 44.53, 37.45, 31.93, 31.79, 30.15, 30.06, 29.01, 28.88, 28.85, 27.57, 26.07, 24.80, 22.77, 18.46, 18.26, 14.21, −3.44, −4.11. HRMS (ESI) calcd for C_31_H_50_O_4_Si [M+H]^+^: *m/z* 515.3551, found: 515.3559.

#### 3.2.9. Synthesis of Compound (**4**)

TBAF (1 M in THF, 3.0 mL, 1.1 equiv) was added to a solution of **9** (1.4 g, 2.72 mmol, 1.0 equiv) in THF (40 mL), and the resultant mixture was stirred at room temperature for 2 h. The reaction was quenched with saturated aq NH_4_Cl (10 mL), and the resultant mixture was extracted with Et_2_O (3 × 10 mL). The organic layers were sequentially washed with water (5 mL) and then with brine (5 mL), and dried over anhydrous MgSO_4_. The solvent of the extract was removed under vacuum, and the residue was subjected to column chromatography on silica gel (hexane/EtOAc: 10/1) to afford **4** (1.0 g, 92%) as a white solid. R_f_ = 0.6 (hexane/EtOAc: 10/1). ^1^H-NMR (600 MHz, chloroform-*d*) δ 7.17 (dt, *J* = 5.1, 2.3 Hz, 1H), 6.40 (d, *J* = 1.8 Hz, 1H), 6.24 (d, *J* = 1.8 Hz, 1H), 3.84 (ddd, *J* = 17.8, 4.8, 2.3 Hz, 1H), 2.68 (td, *J* = 11.3, 4.6 Hz, 1H), 2.50–2.38 (m, 1H), 2.10–1.95 (m, 2H), 1.85 (td, *J* = 11.7, 4.6 Hz, 1H), 1.50 (ddd, *J* = 8.3, 6.2, 3.5 Hz, 2H), 1.42 (s, 3H), 1.26–1.20 (m, 9H), 1.18 (d, *J* = 4.0 Hz, 3H), 1.14 (s, 3H), 1.09–1.01 (m, 2H), 0.85 (t, *J* = 7.1 Hz, 3H). ^13^C-NMR (151 MHz, chloroform-*d*) δ 172.30, 154.64, 154.48, 150.56, 140.54, 130.58, 109.23, 108.05, 105.72, 44.61, 44.14, 37.48, 31.92, 31.17, 30.16, 29.89, 28.91, 28.78, 27.68, 24.75, 22.82, 18.46, 14.24. IR (film, cm^−1^): 3384, 2958, 2927, 2856, 1622, 1412, 1185, 1032, 965, 838. HRMS (ESI) calcd for C_25_H_36_O_4_ [M+H]^+^: *m/z* 401.2686, found: 401.2675.

Δ^8^-THC, ^1^H-NMR (600 MHz, chloroform-*d*) δ 6.29 (d, *J* = 1.6 Hz, 1H), 6.11 (d, *J* = 1.8 Hz, 1H), 5.44 (dd, *J* = 4.8, 2.6 Hz, 1H), 4.66 (s, 1H), 3.24–3.16 (m, 1H), 2.70 (d, *J* = 4.8 Hz, 1H), 2.45 (td, *J* = 7.7, 5.2 Hz, 2H), 2.18–2.11 (m, 1H), 1.89–1.78 (m, 3H), 1.71 (s, 3H), 1.60–1.54 (m, 2H), 1.38 (s, 3H), 1.34–1.29 (m, 4H), 1.11 (s, 3H), 0.89 (t, *J* = 7.0 Hz, 3H). ^13^C-NMR (151 MHz, chloroform-*d*) δ 155.00, 154.88, 142.87, 134.90, 119.47, 110.63, 110.26, 107.75, 45.00, 36.14, 35.57, 31.70, 30.75, 28.02, 27.71, 23.64, 22.69, 18.64, 14.17. HRMS (ESI) calcd for C_21_H_30_O_2_ [M+H]^+^: *m/z* 315.2319, found: 315.2330.

11-nor-Δ^8^-THC-carboxylic acid, ^1^H-NMR (600 MHz, Methanol-*d*_4_) δ 7.03 (dd, *J* = 5.2, 2.6 Hz, 1H), 6.18 (d, *J* = 1.6 Hz, 1H), 6.10 (d, *J* = 1.6 Hz, 1H), 3.87 (ddd, *J* = 17.7, 4.5, 2.4 Hz, 1H), 2.60 (td, *J* = 11.2, 4.4 Hz, 1H), 2.43 (d, *J* = 8.6 Hz, 1H), 2.40 (d, *J* = 7.7 Hz, 2H), 2.10–1.96 (m, 1H), 1.85–1.72 (m, 2H), 1.59–1.53 (m, 2H), 1.37 (s, 3H), 1.36–1.28 (m, 4H), 1.09 (s, 3H), 0.91 (t, *J* = 7.1 Hz, 3H). ^13^C-NMR (151 MHz, Methanol-*d*_4_) δ 170.90, 157.83, 155.76, 143.73, 139.41, 132.43, 110.98, 109.75, 108.54, 77.00, 46.06, 36.62, 32.80, 32.66, 32.05, 31.52, 29.55, 27.91, 23.60, 18.43, 14.40. HRMS (ESI) calcd for C_21_H_28_O_4_ [M+H]^+^: *m/z* 345.2060, found: 345.2056.

Δ^9^-THC, ^1^H-NMR (600 MHz, chloroform-*d*) δ 6.33–6.30 (m, 1H), 6.28 (d, *J* = 1.6 Hz, 1H), 6.15 (d, *J* = 1.6 Hz, 1H), 4.80 (s, 1H), 3.21 (dt, *J* = 10.9, 2.6 Hz, 1H), 2.44 (td, *J* = 7.5, 3.5 Hz, 2H), 2.21–2.14 (m, 2H), 1.95–1.89 (m, 1H), 1.70 (d, *J* = 2.0 Hz, 1H), 1.69 (dq, *J* = 2.3, 1.0 Hz, 3H), 1.59–1.54 (m, 2H), 1.42 (s, 3H), 1.41 (s, 1H), 1.33–1.28 (m, 4H), 1.10 (s, 3H), 0.90–0.87 (m, 3H). ^13^C-NMR (151 MHz, chloroform-*d*) δ 154.91, 154.30, 142.97, 134.56, 123.85, 110.23, 109.17, 107.68, 77.37, 45.93, 35.61, 33.70, 31.65, 31.30, 30.79, 27.71, 25.15, 23.51, 22.68, 19.41, 14.16. HRMS (ESI) calcd for C_21_H_30_O_2_ [M+H]^+^: *m/z* 315.2319, found: 315.2320.

Δ^9^-THC-carboxylic acid, ^1^H-NMR (400 MHz, Methanol-*d*_4_) δ 8.05 (d, *J* = 2.2 Hz, 1H), 6.20 (s, 1H), 6.11 (s, 1H), 3.35 (d, *J* = 3.4 Hz, 1H), 2.53 (dd, *J* = 18.5, 6.8 Hz, 1H), 2.42 (t, *J* = 7.6 Hz, 3H), 2.04 (dd, *J* = 12.8, 7.3 Hz, 1H), 1.69–1.51 (m, 3H), 1.41 (s, 4H), 1.32 (ddd, *J* = 12.7, 10.0, 5.6 Hz, 4H), 1.09 (s, 3H), 0.90 (t, *J* = 6.9 Hz, 3H). ^13^C-NMR (151 MHz, Methanol-*d*_4_) δ 171.52, 157.20, 155.95, 144.78, 144.07, 130.08, 109.84, 108.39, 108.19, 77.92, 46.15, 36.64, 35.95, 32.65, 32.06, 27.92, 26.66, 25.50, 23.61, 19.25, 14.40. HRMS (ESI) calcd for C_21_H_28_O_4_ [M+H]^+^: *m/z* 345.2060, found: 345.2059.

## 4. Conclusions

In this study, a straightforward synthetic route was developed for the key intermediate for the synthesis of various cannabinoid metabolites on a multigram scale. The optimization of the Riley oxidation of the key tricyclic intermediate significantly improved the yield and made it possible to apply this condition to the synthesis of different analogues of this skeleton. This kind of analogue can be used as a product to meet the diverse needs of various laboratories for the synthesis of potential cannabinoid metabolites. Similar synthesis strategies could be applied for the synthesis of similar metabolites of other cannabinoid metabolites like 11-nor-Δ^8^-THC-carboxylic acid and Δ^9^-THC-carboxylic acid.

## Figures and Tables

**Table 1:** The optimization of the Riley oxidation.

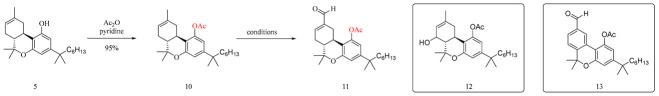				
Entry	Substrate	Condition	Product	Yield ^a^
1	5	SeO_2_, DCM	11	15%
2	5	SeO_2_, THF, H_2_O, 65 °C	11	25%
3	5	SeO_2_, *t*BuOOH, DCM	11	8%
4	5	SeO_2_, *t*BuOOH, salicylic acid	11	12%
5	5	SeO_2_, AcOH, dioxane	11	0%
6	5	SeO_2_, dioxane, 110 °C	11	39%
7	6	SeO_2_, dioxane, 110 °C	7 ^b^	65%

^a^ Isolated yield. ^b^ Spectrums can be found in Supplementary Materials.

## Data Availability

Data are contained within the article and supplementary materials.

## Supplementary Materials

The following supporting information can be downloaded at: https://www.mdpi.com/article/10.3390/molecules29020526/s1. Figures S1–S31: NMR and HRMS spectra for eleven products (compound **5**, compound **6**, compound **7**, compound **8**, compound **10**, compound **11**, compound **12**, compound **14**, compound **15**, compound **3**, compound **9**, compound **4**, compound **12**, Δ^8^-THC, Δ^9^-THC, 11-nor-Δ^8^-THC-carboxylic acid, Δ^9^-THC-carboxylic acid).
