# Supplementary material for: Syntheses of Cannabinoid Metabolites: Ajulemic Acid and HU-210
Source: Molecules. 2024 Jan 21;29(2):526. doi: 10.3390/molecules29020526 (PMC10818984; doi:10.3390/molecules29020526)
Supplement: Supplementary file 1 [file molecules-29-00526-s001.zip › molecules-2806847-supplementary.pdf]

Supporting Information

Synthesis of cannabinoid metabolites: ajulemic acid and HU-210

Wenbin Shao[1,2], Pingyong Liao[1,2], Xiaoyan Zhang[3], Binbin Fan[3], Ruijia Chen[1,2], Xilong Chen[1,2], Xuejun Zhao[1,2,*], Wenbin Liu[1,2,*]

[1] Shanghai Key Laboratory of Crime Scene Evidence, Shanghai Research Institute of Criminal Science and Technology, Shanghai 200072, China

[2] Shanghai Yuansi Standard Science and Technology Co., Ltd, Shanghai, 200072, China

[3] State Key Laboratory of Bio-Fibers and Eco-Textiles & Institute of Marine Biobased Materials & Collage of Materials Science and Engineering, Qingdao University, Qingdao 266071, China

* Correspondence: wbliu1981@163.com (W.L.); xjzhao1201@163.com (X.Z.)

Table of contents

Figure S1-S2. NMR spectra of intermediate 5.
Figure S3-S4. NMR spectra of intermediate 6.
Figure S5-S6. NMR spectra of intermediate 7.
Figure S7-S8. NMR spectra of intermediate 8.
Figure S9-S10. NMR spectra of intermediate 10.
Figure S11-S12. NMR spectra of intermediate 11.
Figure S13-S14. NMR spectra of intermediate 12.
Figure S15-S16. NMR spectra of intermediate 14.
Figure S17. ^{1}HNMR spectra of intermediate 15.
Figure S18-S19. NMR spectra of intermediate 3.
Figure S20-S21. NMR spectra of intermediate 9.
Figure S22-S23. NMR spectra of intermediate 4.
Figure S24-S25. NMR spectra of intermediate Δ^8-THC.
Figure S26-S27. NMR spectra of intermediate 11-Nor-Δ^8-THC-carboxylic acid.
Figure S28-S29. NMR spectra of intermediate Δ^9-THC.
Figure S30-S31. NMR spectra of intermediate Δ^9-THC- carboxylic acid.

NMR spectra for the synthesized compounds

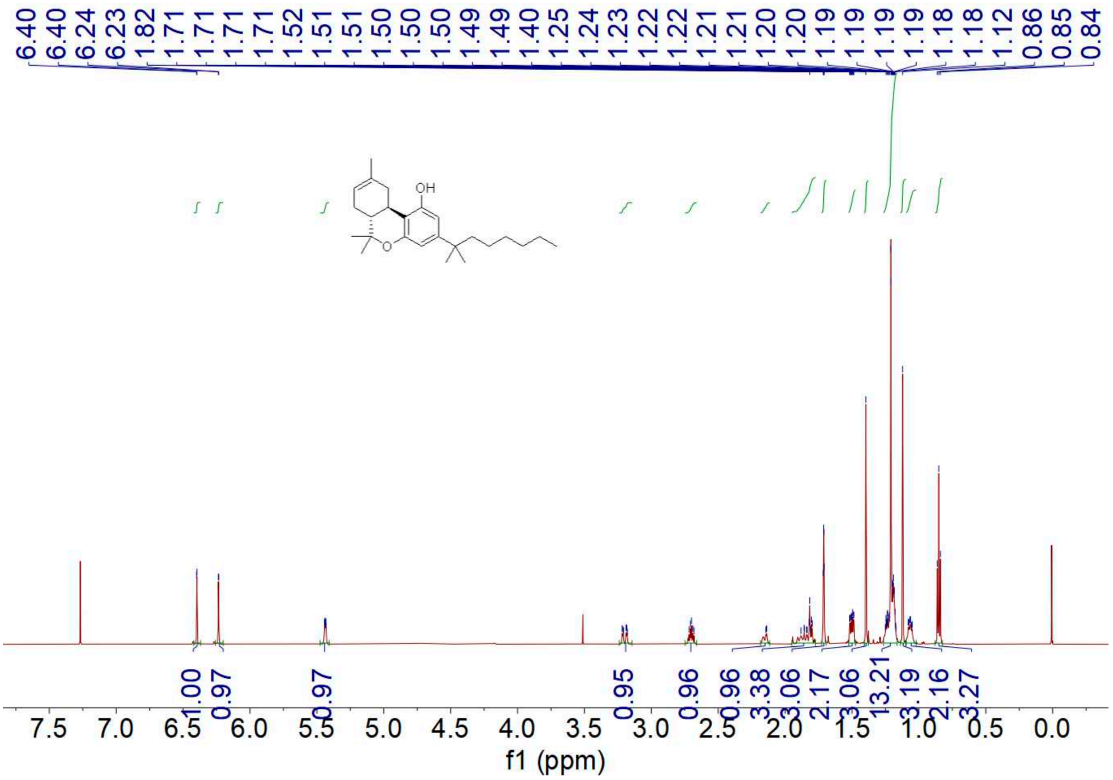

Figure S1 ^{1}H-NMR of compound **5** ($CDCl_3$)

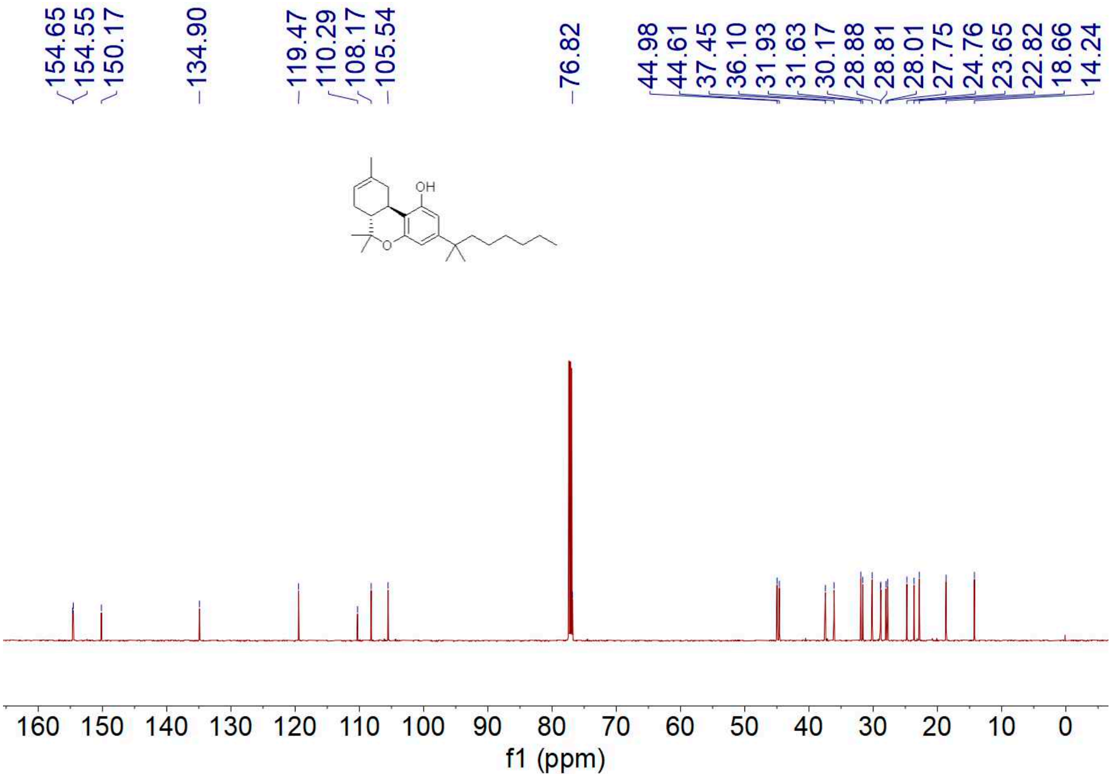

Figure S2 ^{13}C-NMR of compound **5** ($CDCl_3$)

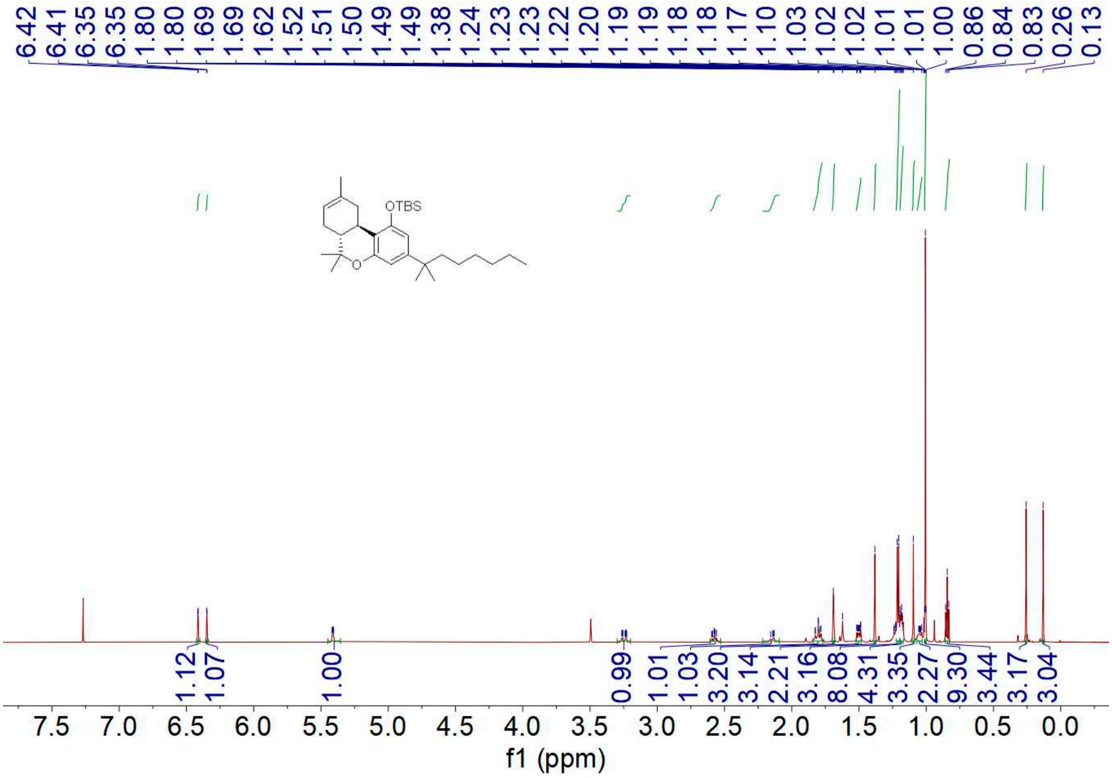

Figure S3 ^{1}H-NMR of compound **6** ($CDCl_3$)

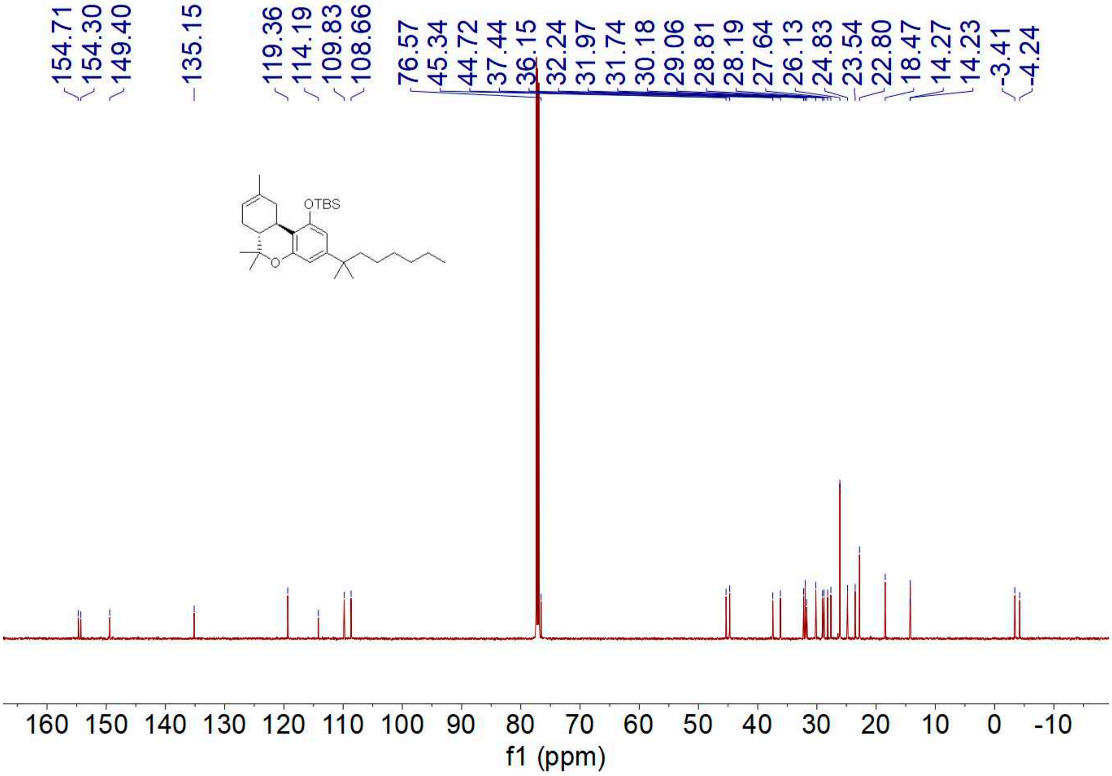

Figure S4 ^{13}C-NMR of compound **6** ($CDCl_3$)

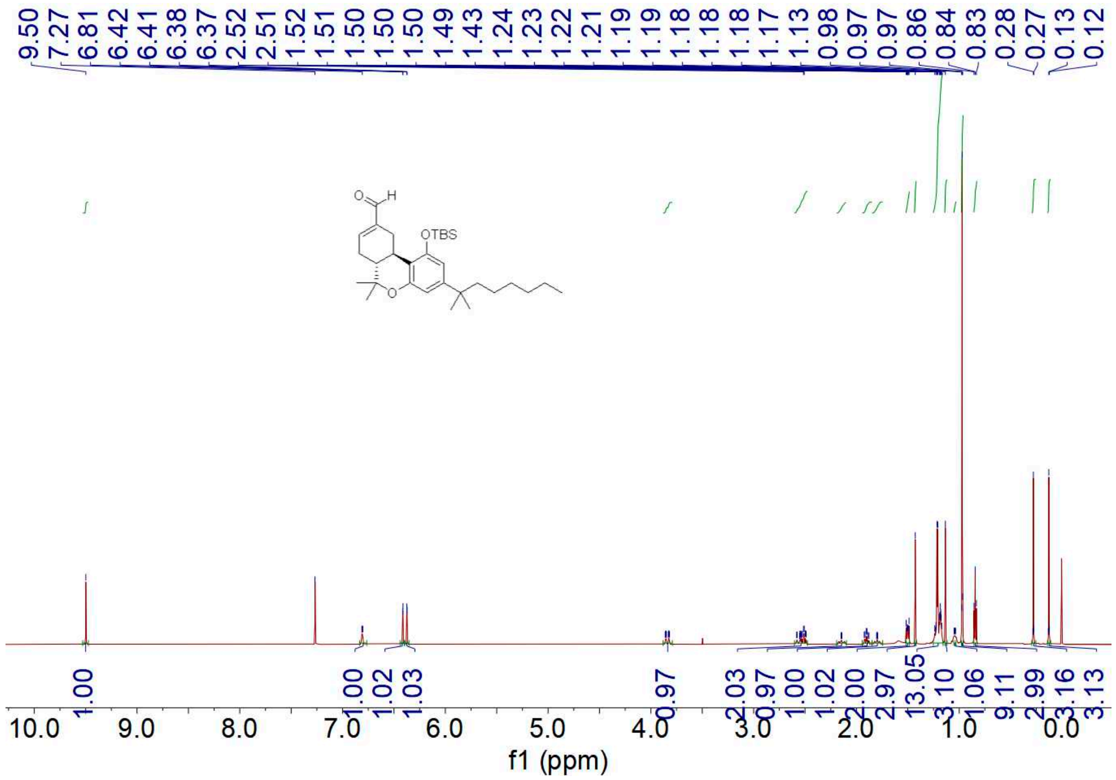

Figure S5 ^{1}H-NMR of compound **7** ($CDCl_3$)

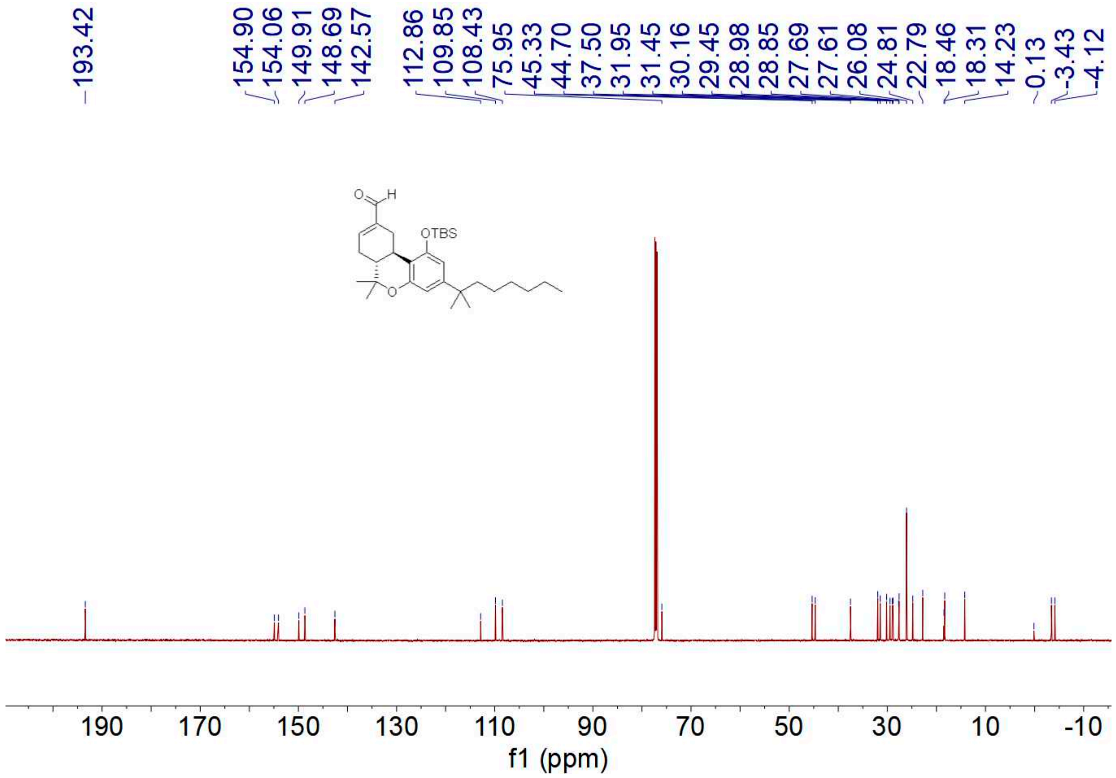

Figure S6 ^{13}C-NMR of compound **7** ($CDCl_3$)

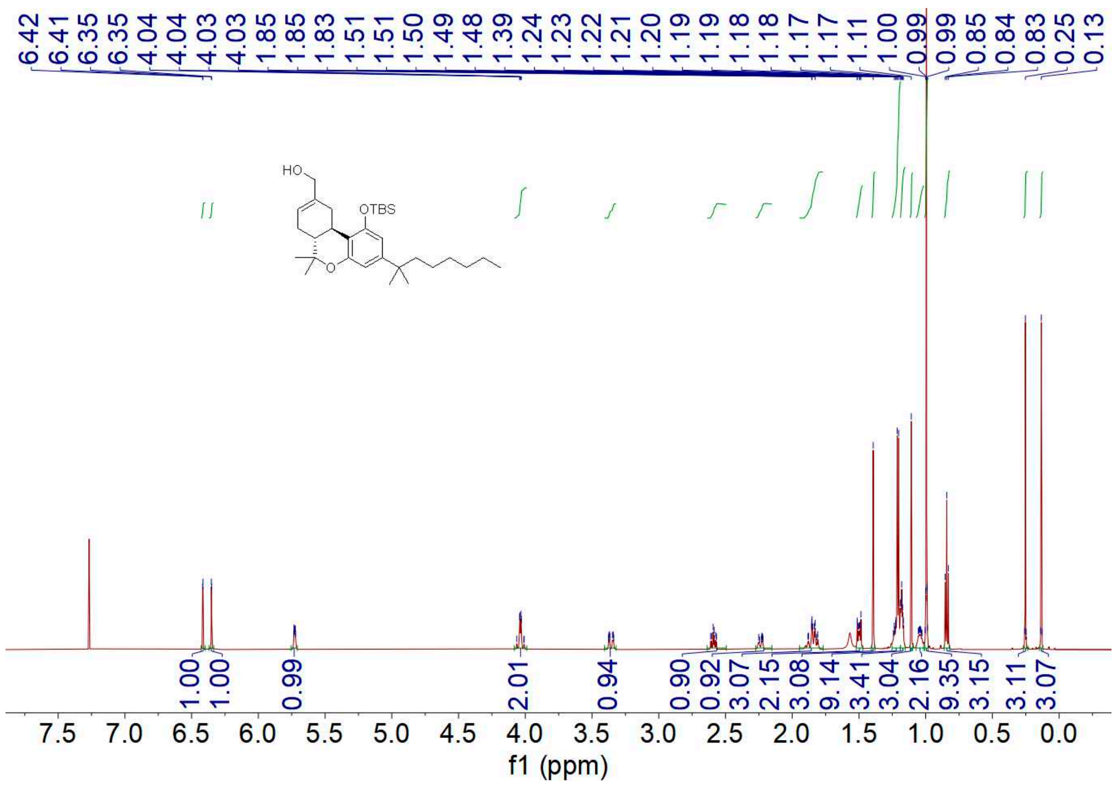

Figure S7 ^{1}H-NMR of compound **8** ($CDCl_3$)

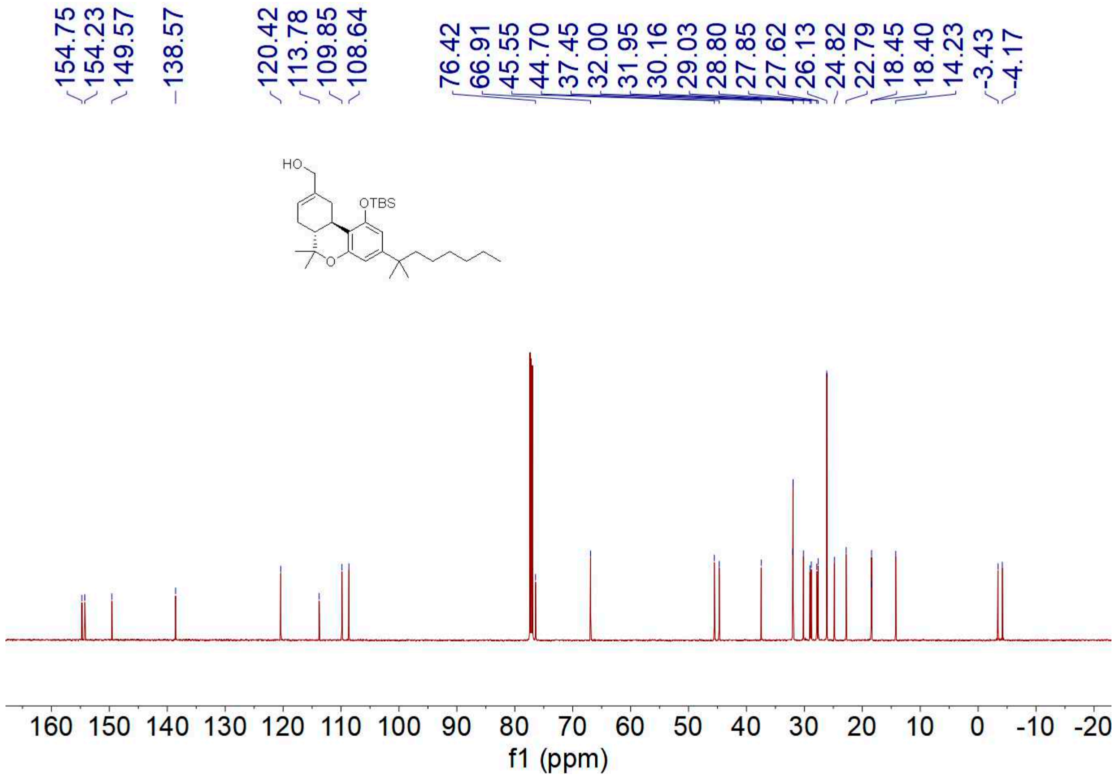

Figure S8 ^{13}C-NMR of compound **8** ($CDCl_3$)

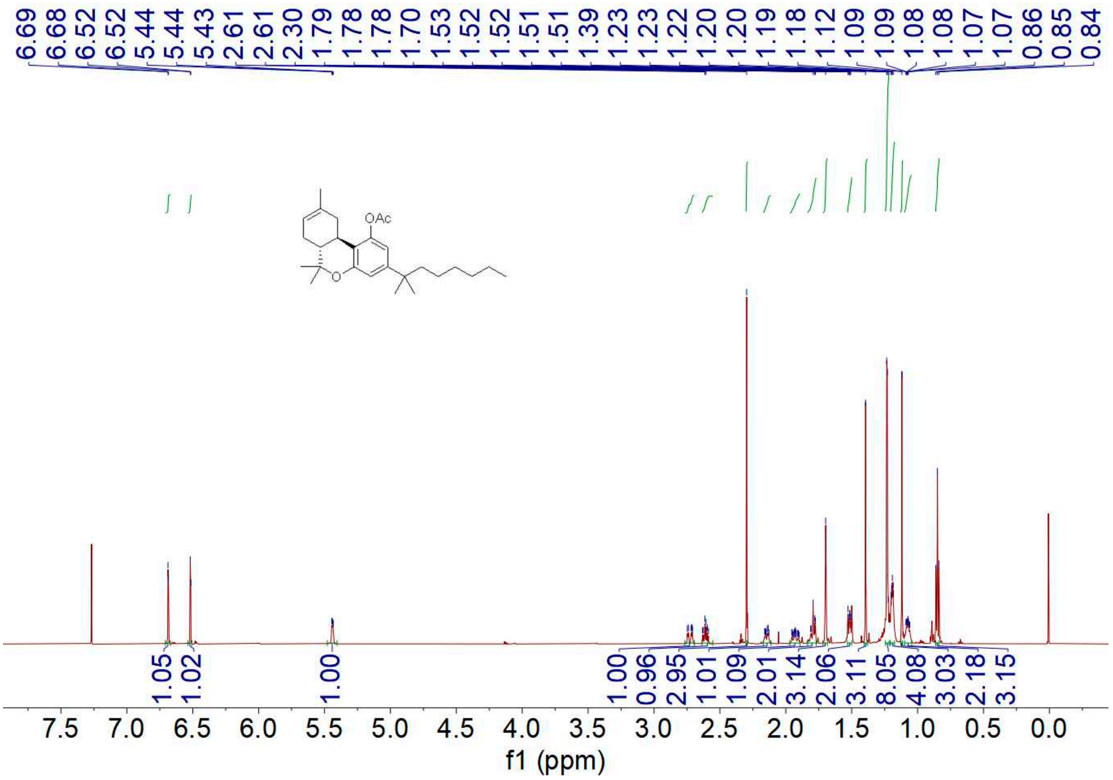

Figure S9 ^{1}H-NMR of compound **10** ($CDCl_3$)

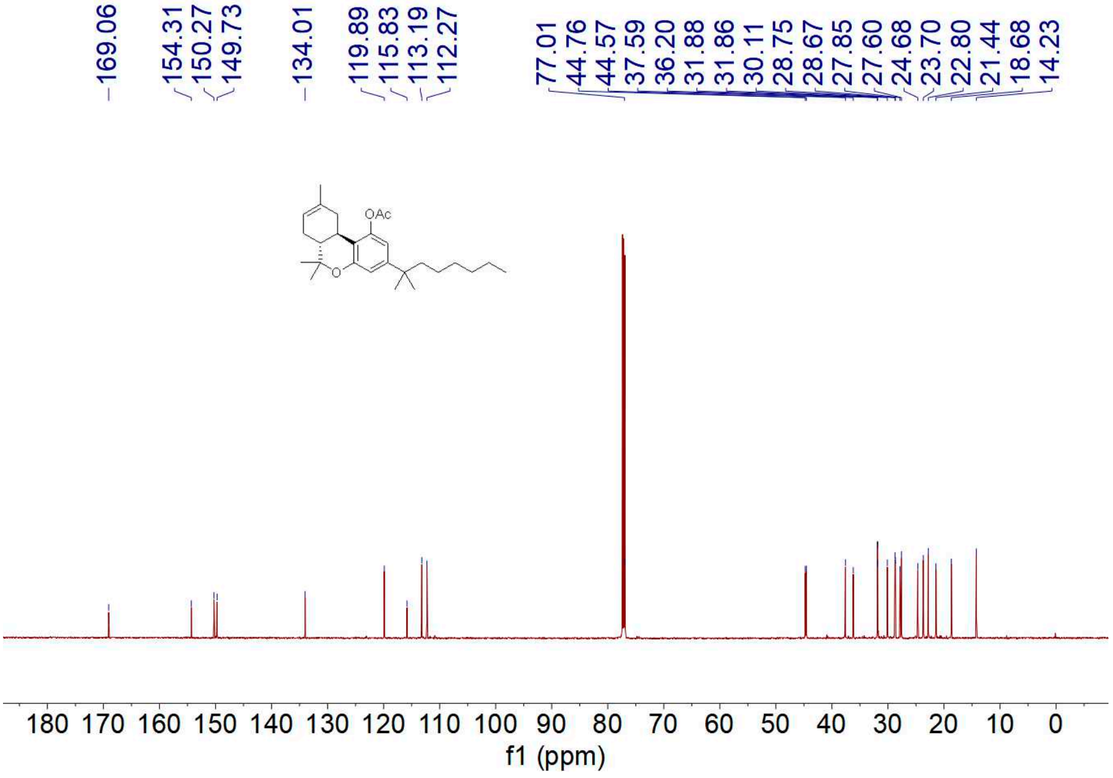

Figure S10 ^{13}C-NMR of compound **10** ($CDCl_3$)

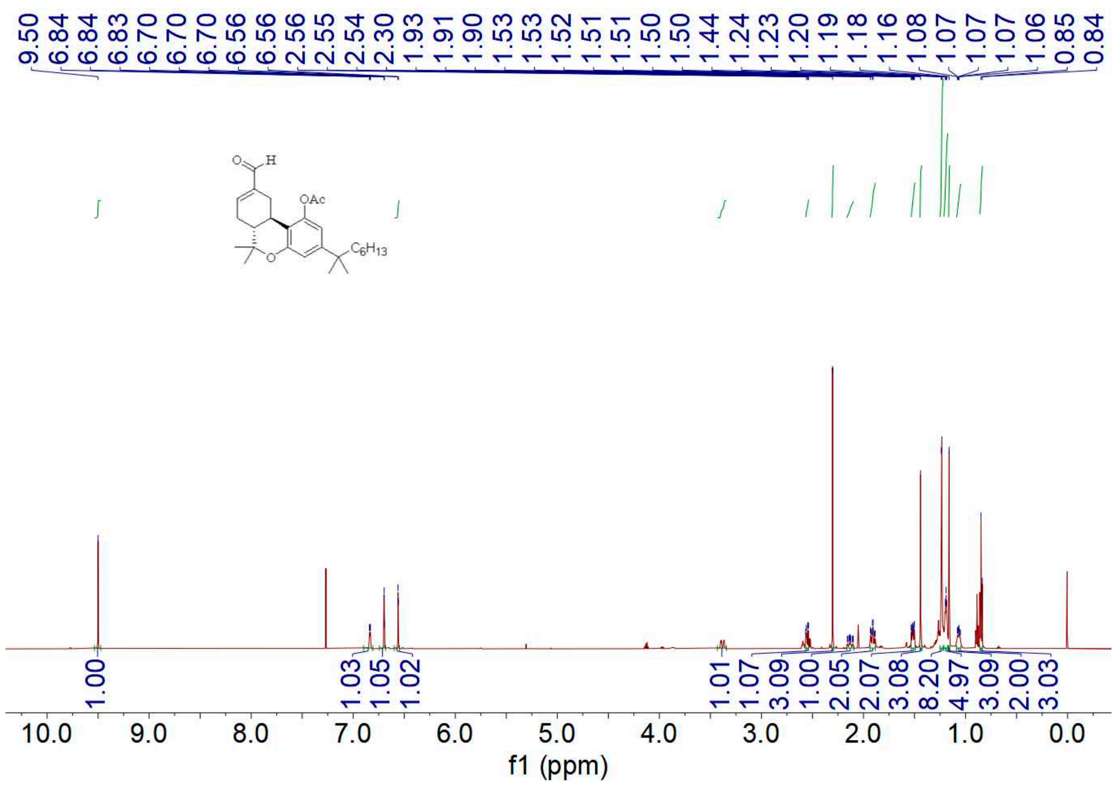

Figure S11 ^{1}H-NMR of compound **11** ($CDCl_3$)

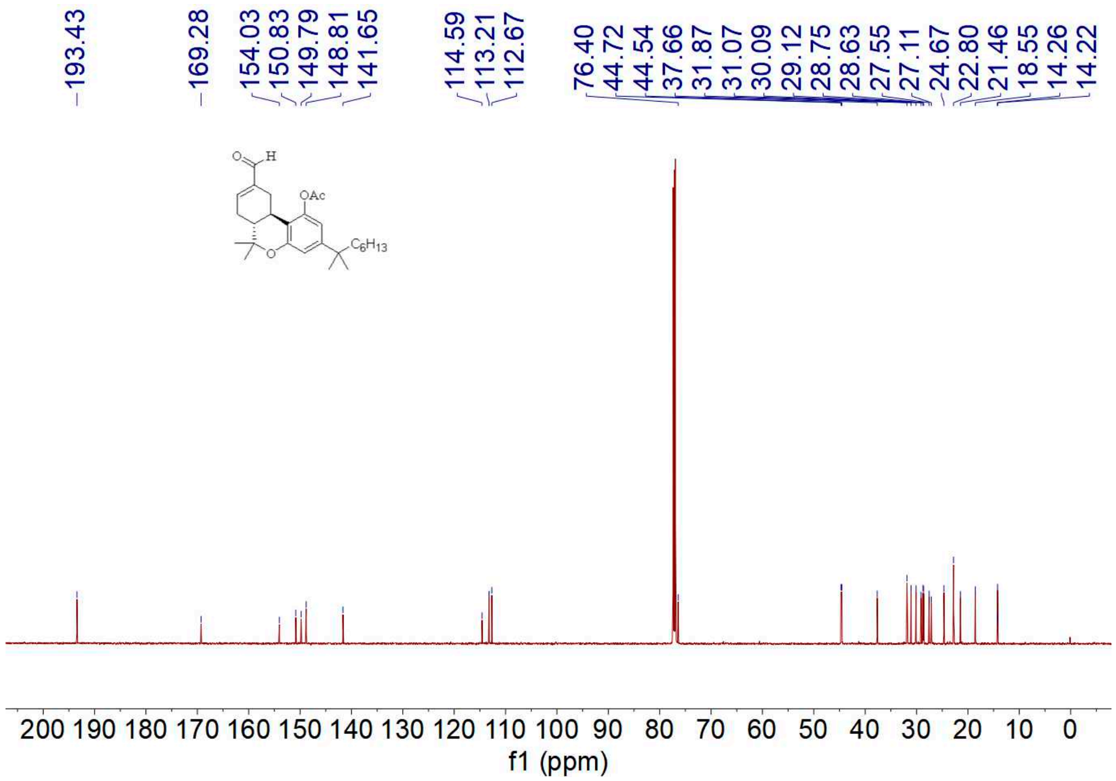

Figure S12 ^{13}C-NMR of compound **11** ($CDCl_3$)

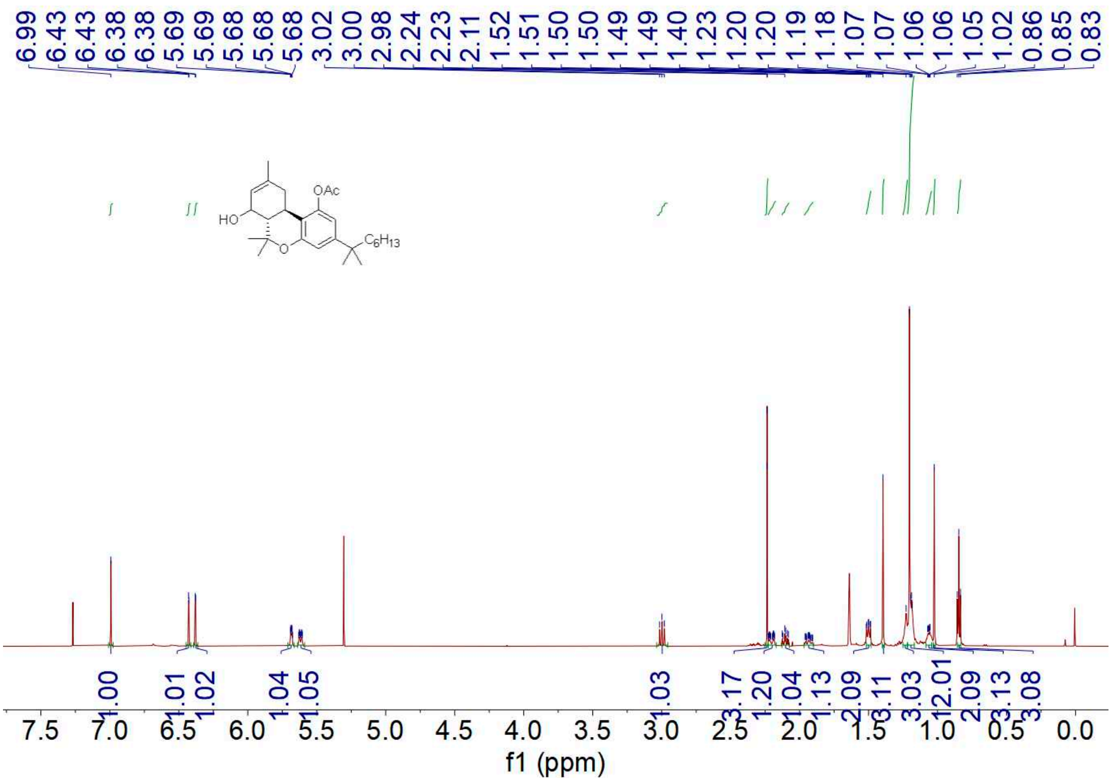

Figure S13 ^{1}H-NMR of compound **12** ($CDCl_3$)

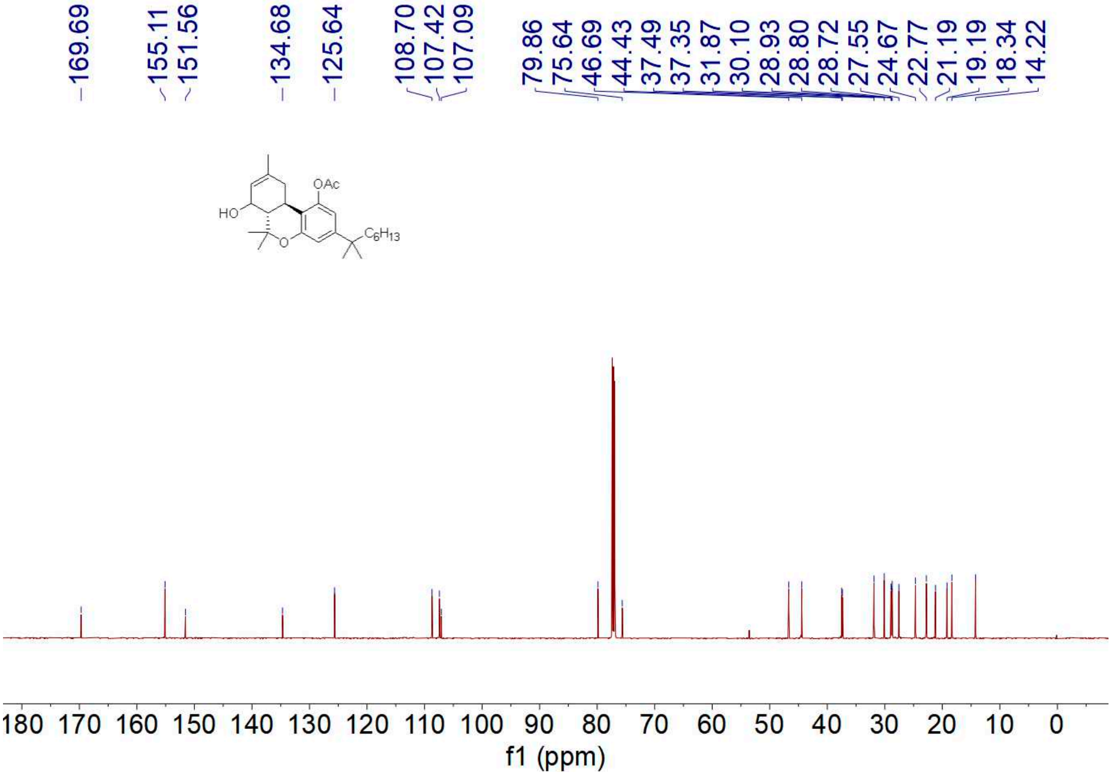

Figure S14 ^{13}C-NMR of compound **12** ($CDCl_3$)

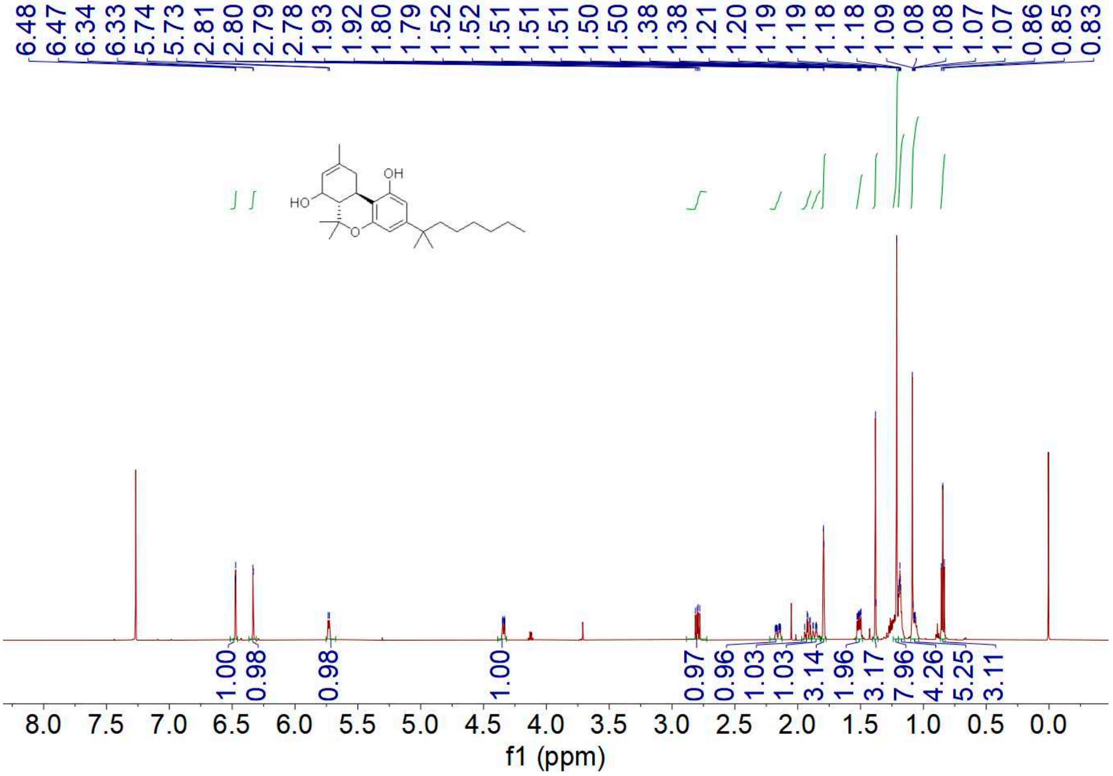

Figure S15 ^{1}H-NMR of compound **14** ($CDCl_3$)

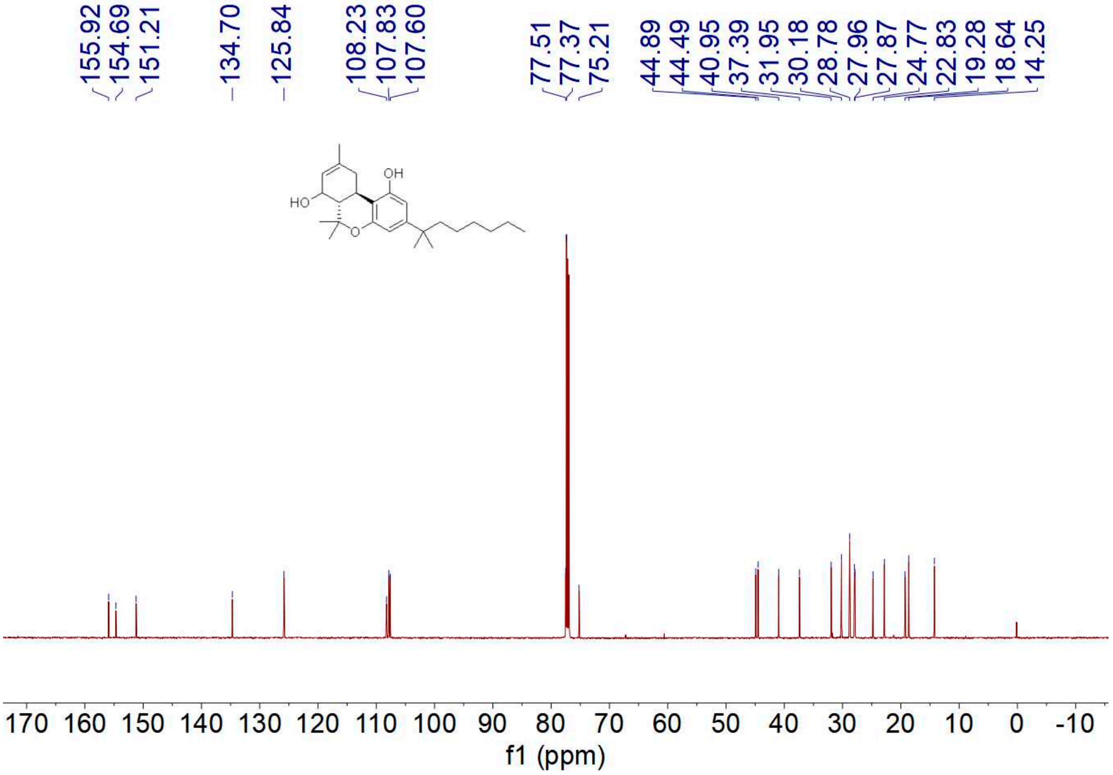

Figure S16 ^{13}C-NMR of compound **14** ($CDCl_3$)

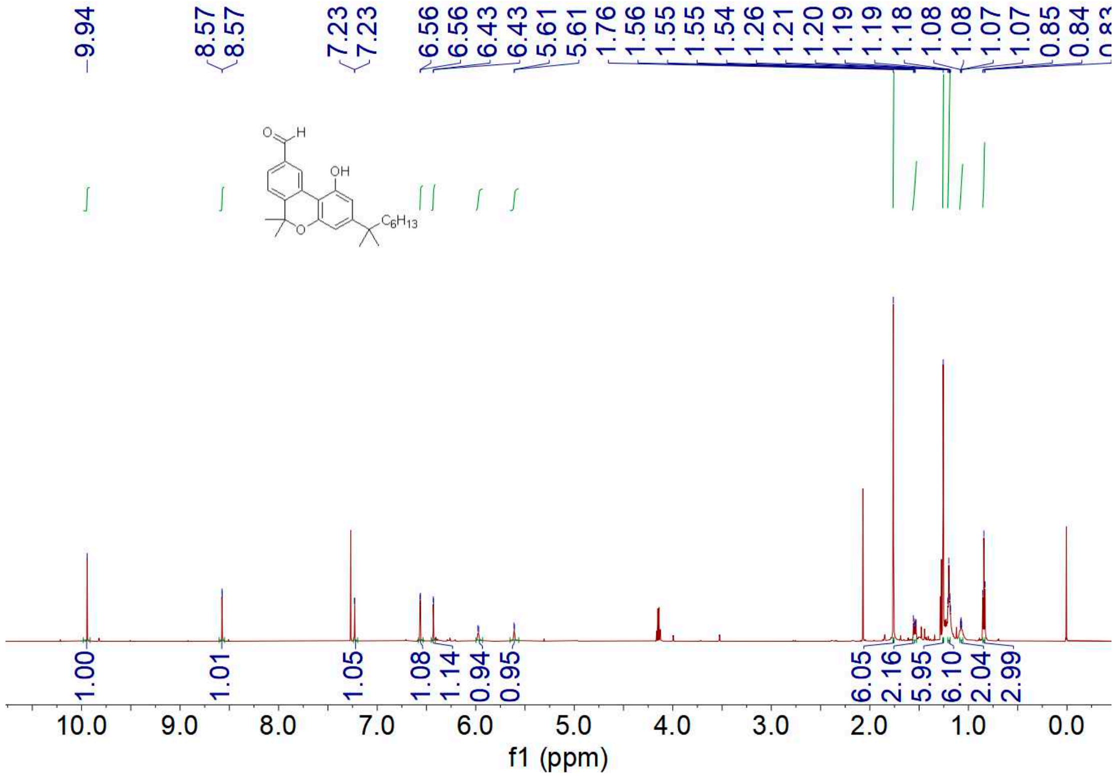

Figure S17 ^{1}H-NMR of compound **15** ($CDCl_3$)

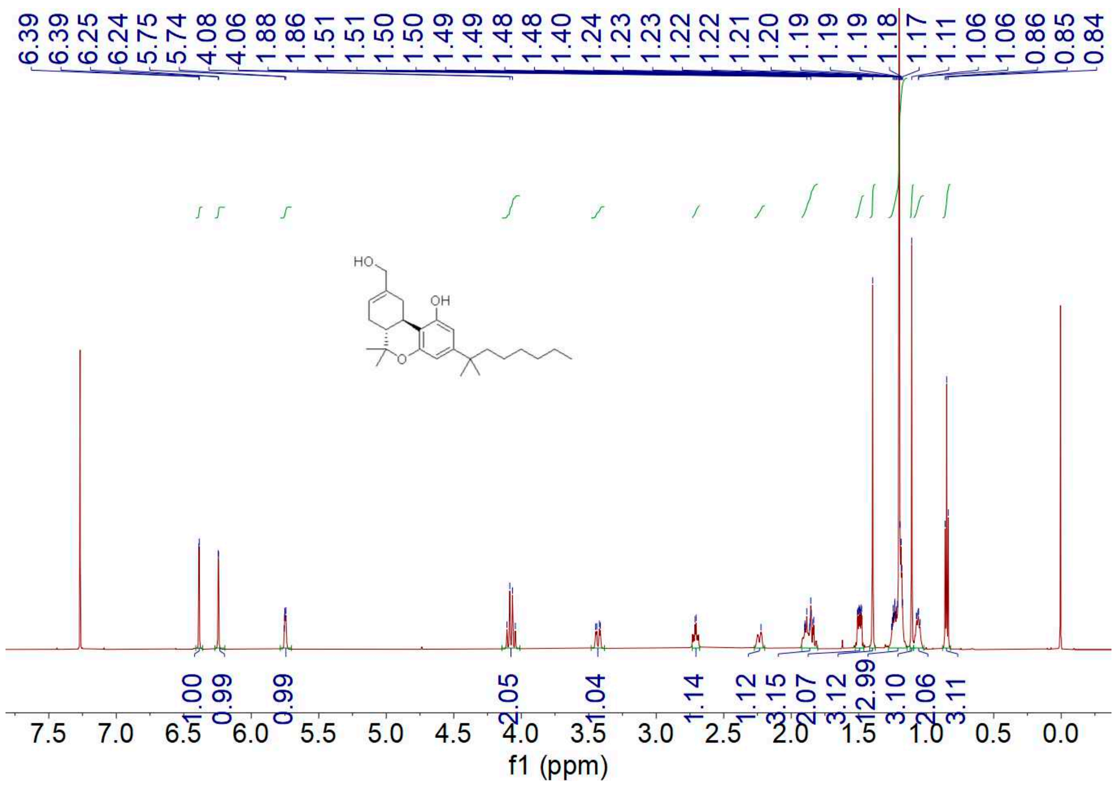

Figure S18 ^{1}H-NMR of compound **3** ($CDCl_3$)

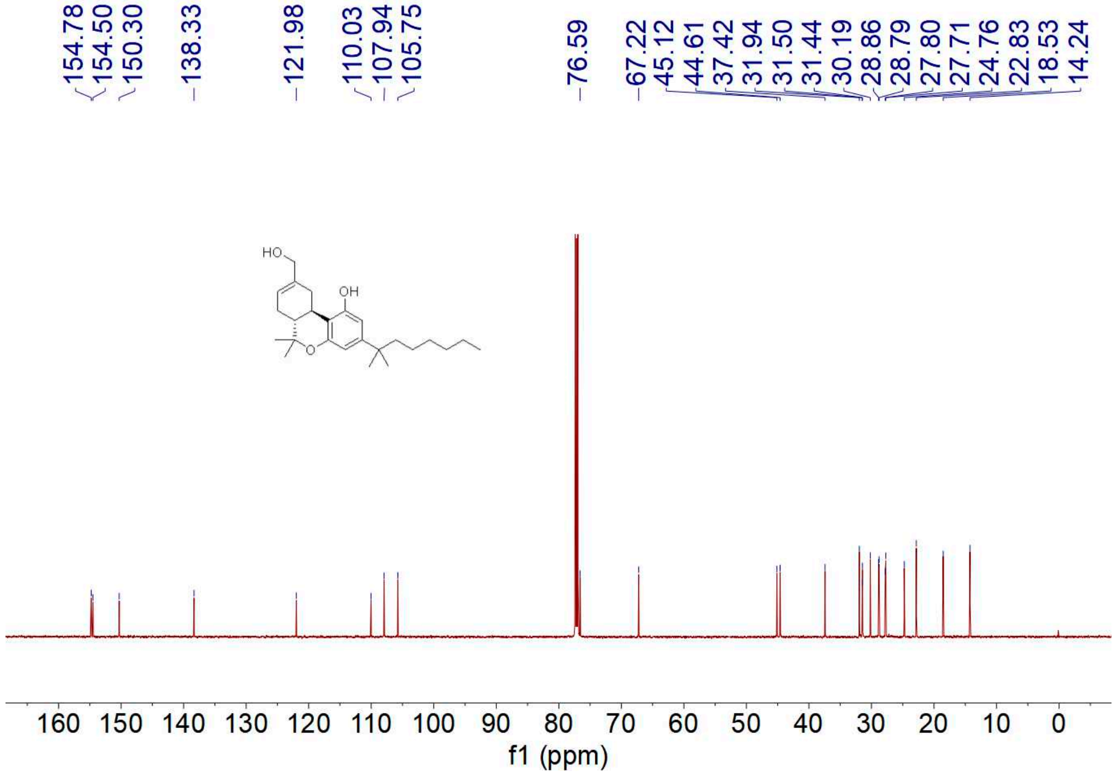

Figure S19 ^{13}C-NMR of compound **3** ($CDCl_3$)

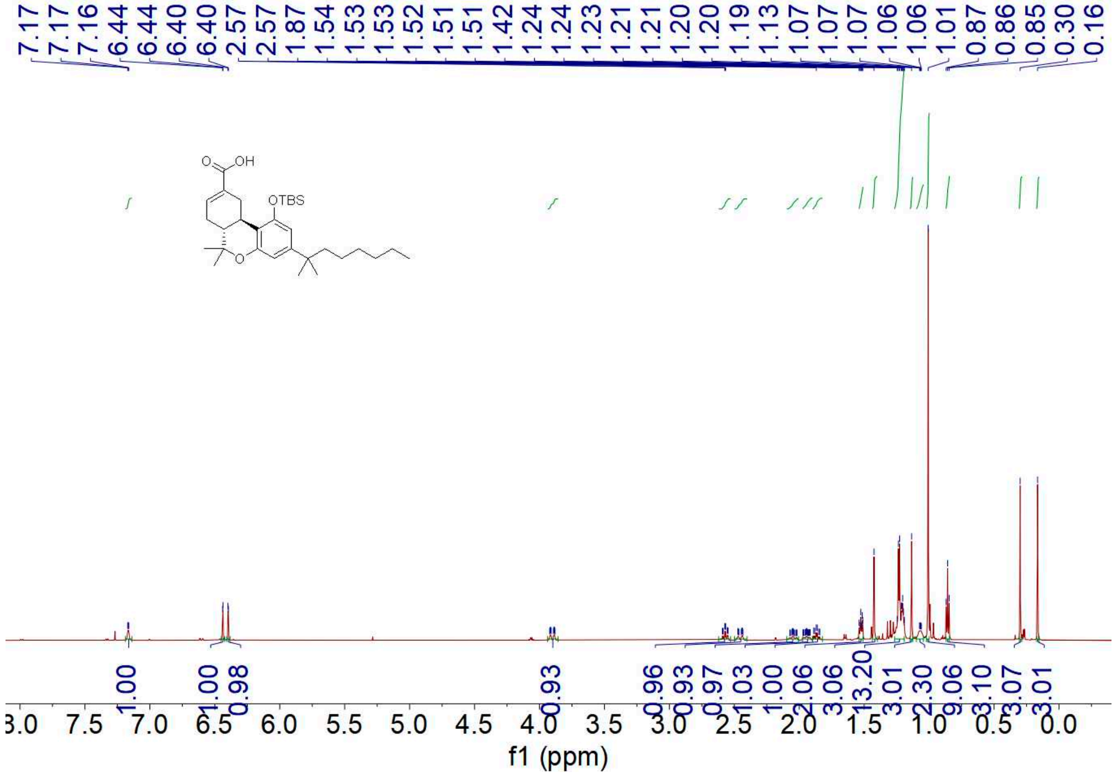

Figure S20 ^{1}H-NMR of compound **9** ($CDCl_3$)

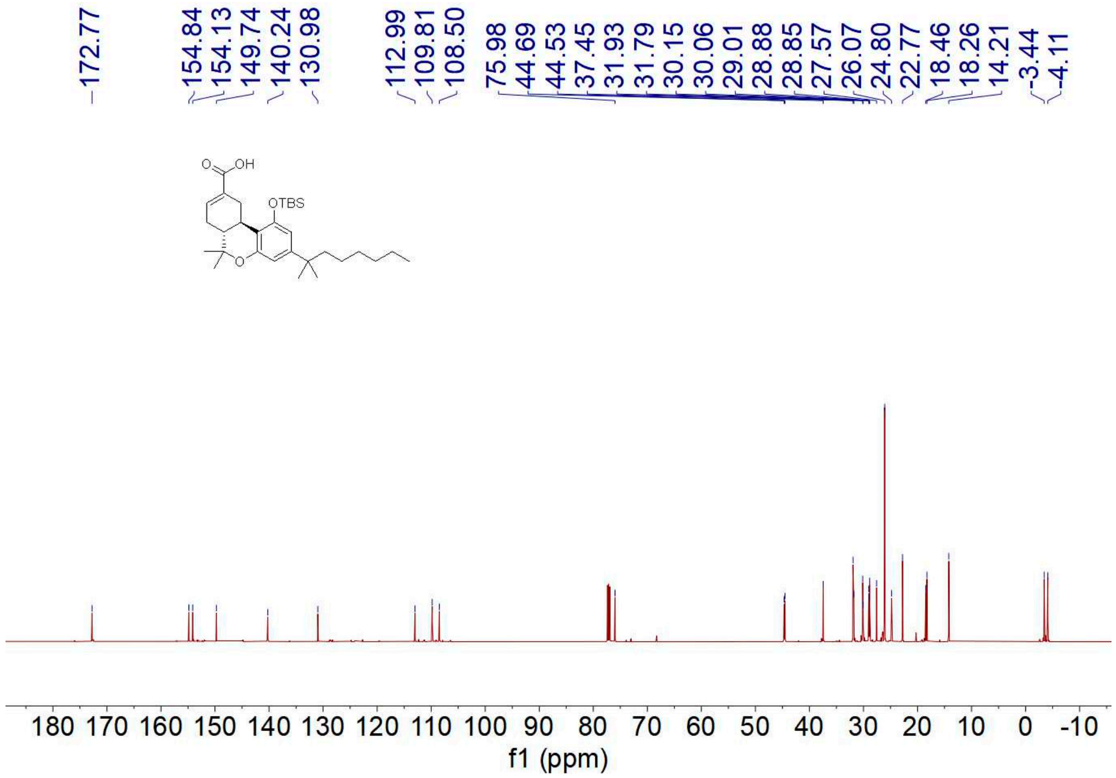

Figure S21 ^{13}C-NMR of compound **9** ($CDCl_3$)

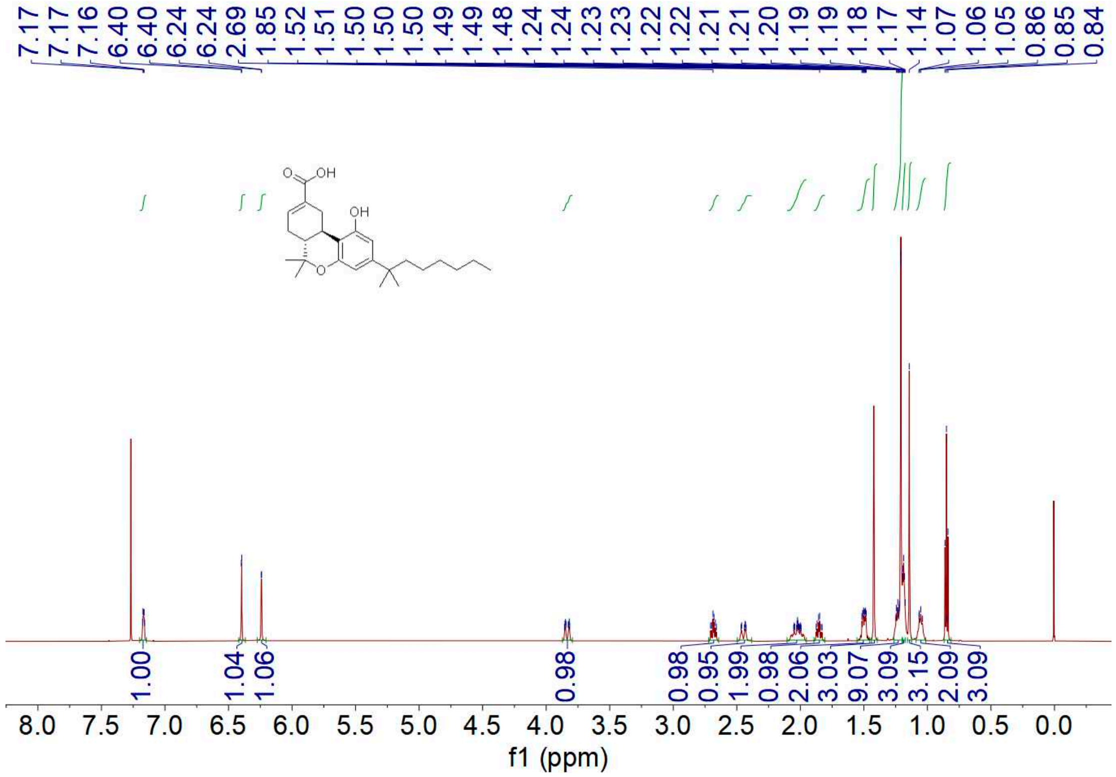

Figure S22 ^{1}H-NMR of compound **4** ($CDCl_3$)

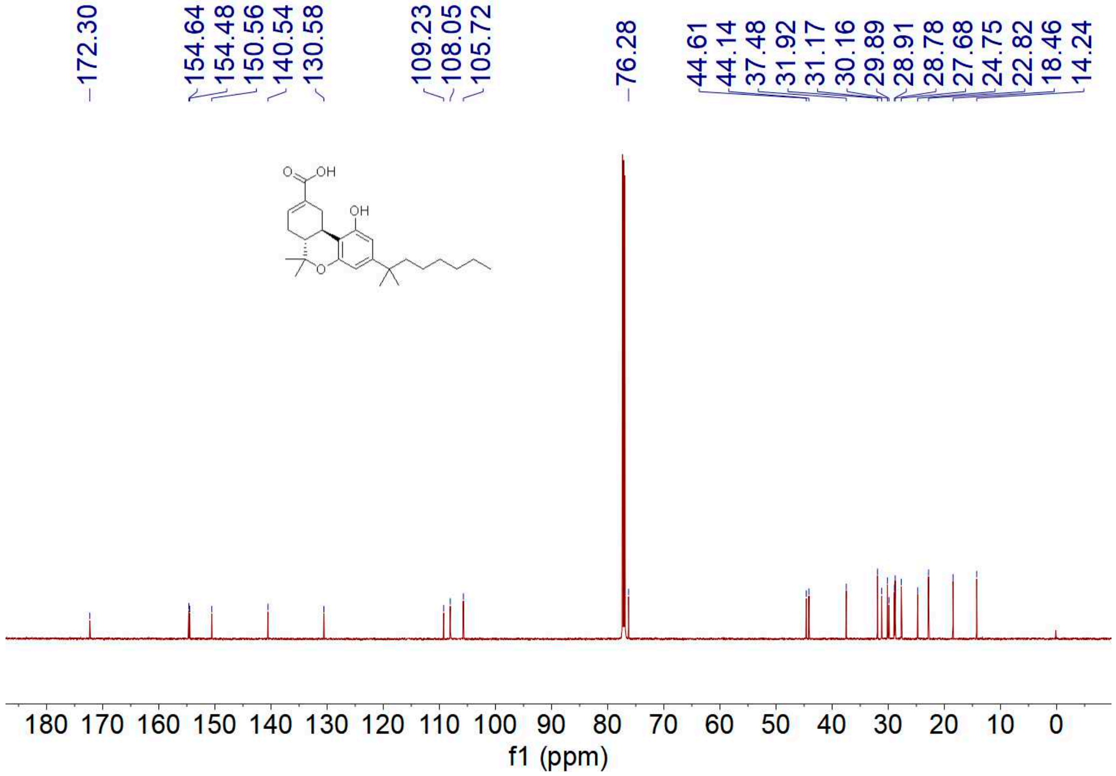

Figure S23 ^{13}C-NMR of compound **4** ($CDCl_3$)

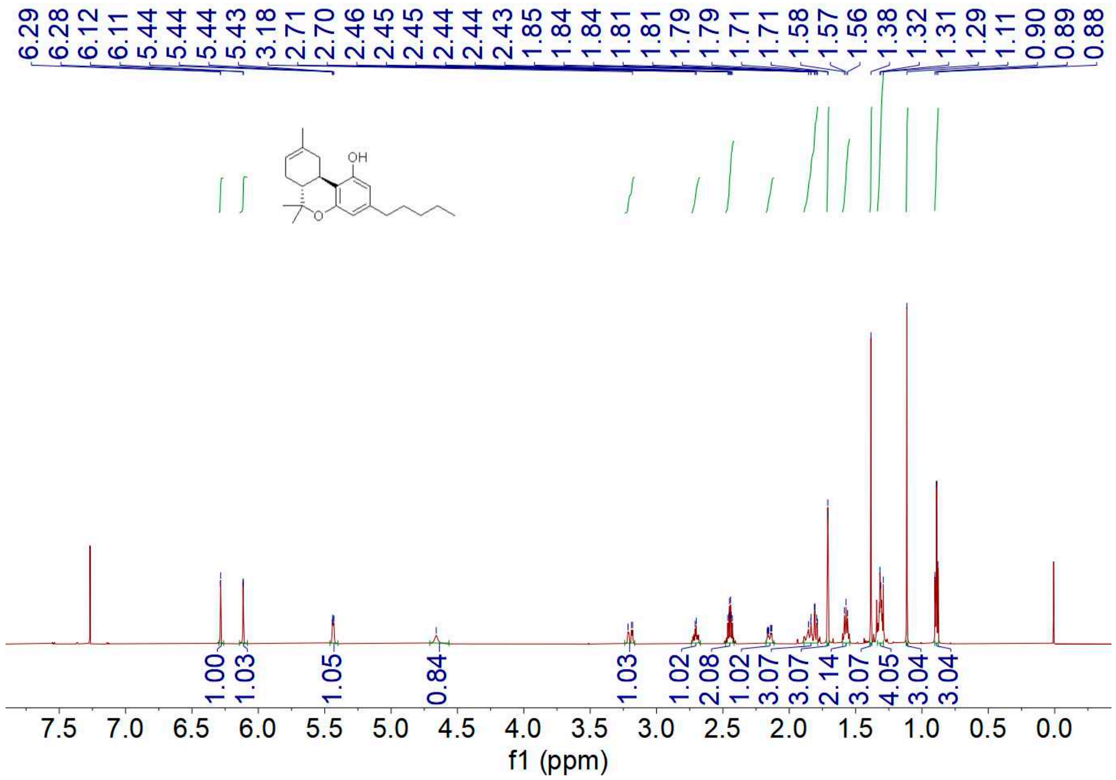

Figure S24 ^{1}H-NMR of compound Δ^{8}-THC ($CDCl_3$)

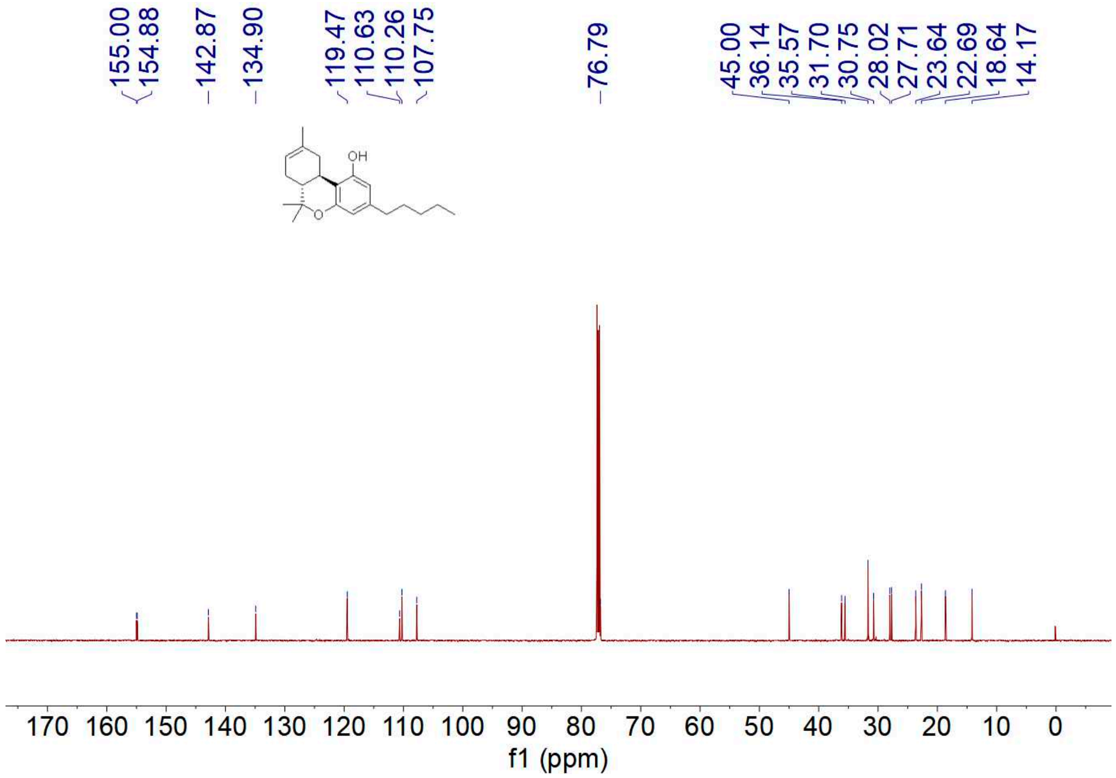

Figure S25 ^{13}C-NMR of compound Δ^{8}-THC ($CDCl_3$)

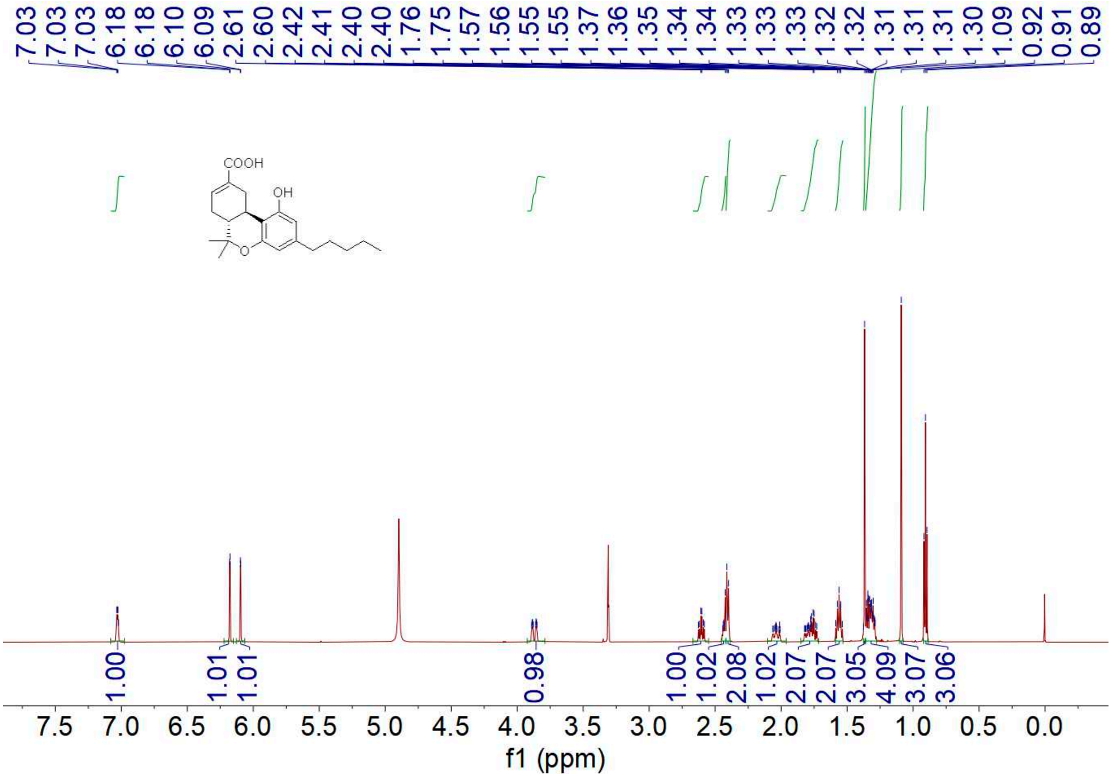

Figure S26 ^{1}H-NMR of compound 11- nor-Δ^{8}-THC-carboxylic acid (CD_3OD)

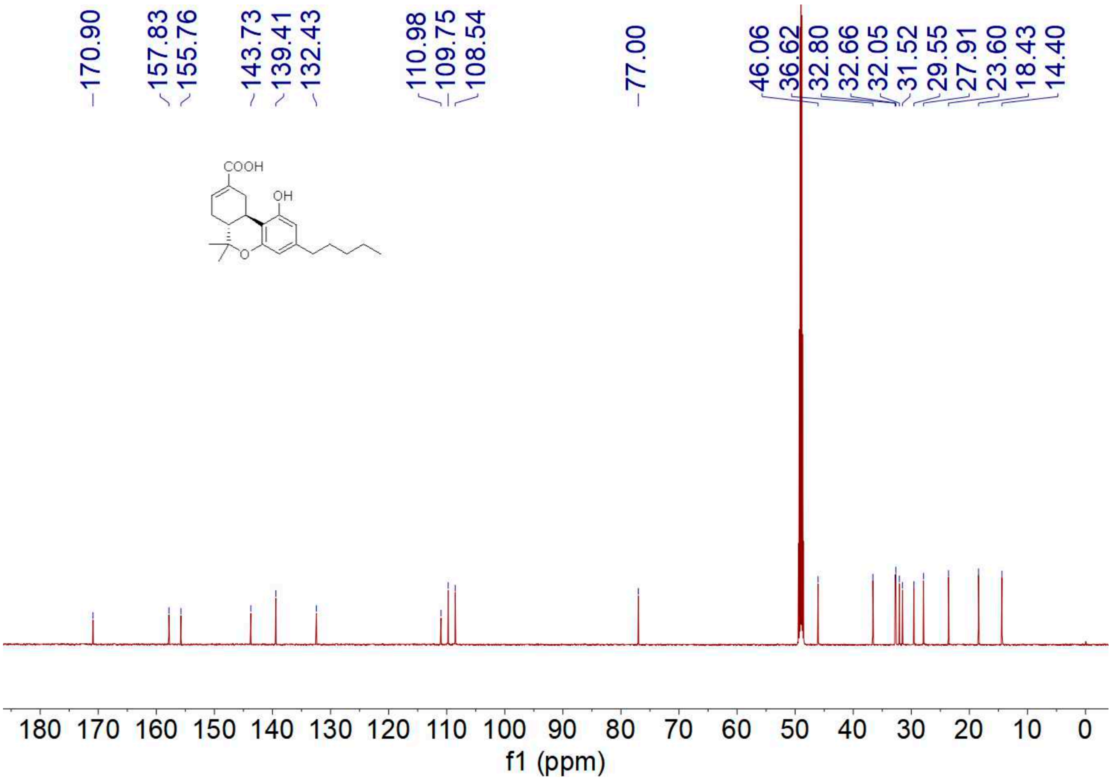

Figure S27 ^{13}C-NMR of compound 11-nor-Δ^{8}-THC-carboxylic acid (CD_3OD)

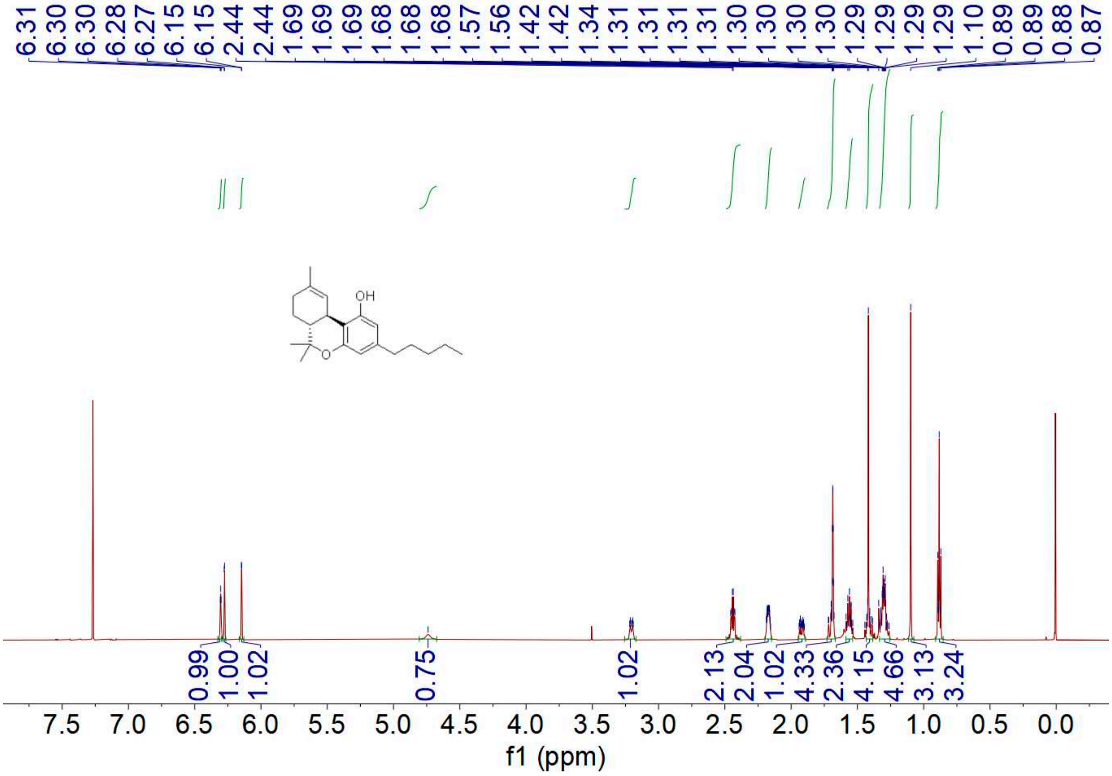

Figure S28 ^{1}H-NMR of compound Δ^{9}-THC ($CDCl_3$)

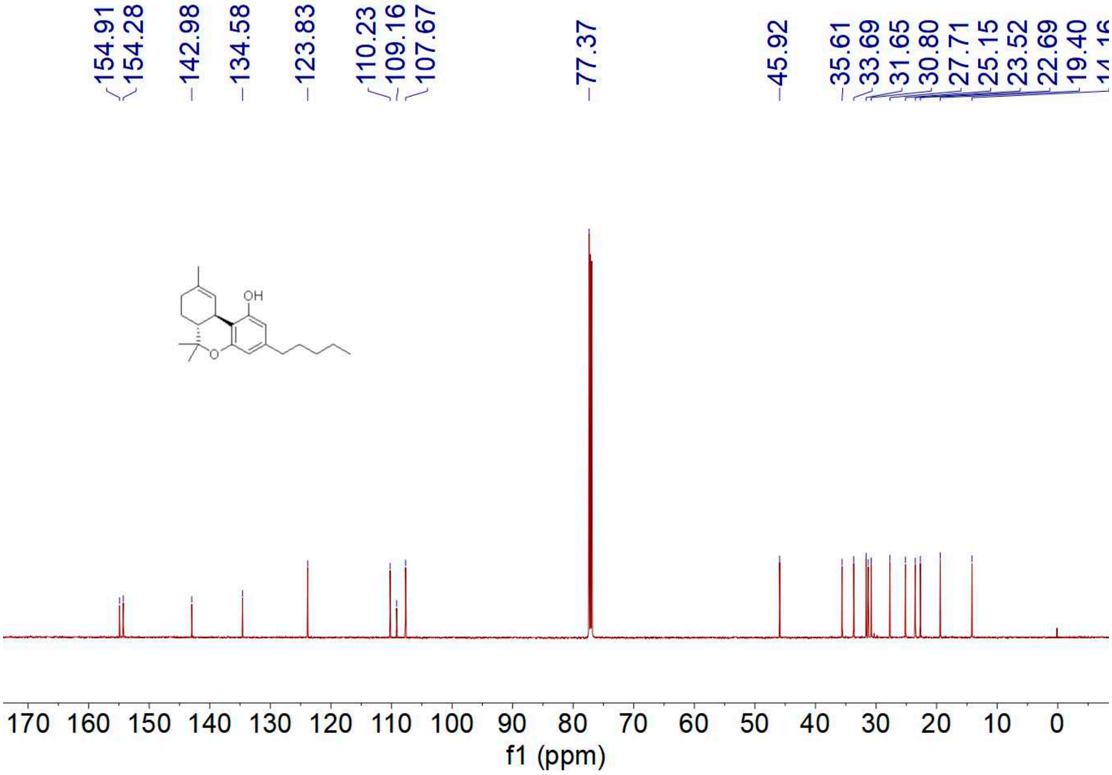

Figure S29 ^{13}C-NMR of compound Δ^{9}-THC ($CDCl_3$)

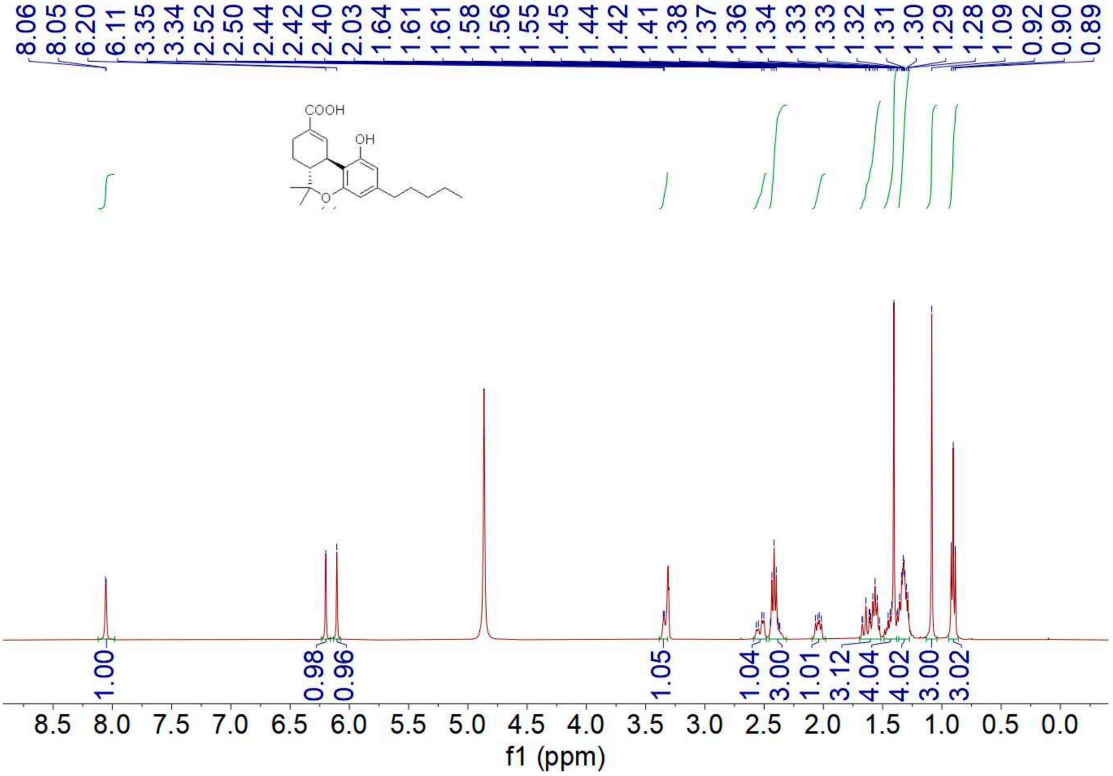

Figure S30 ^{1}H-NMR of compound Δ^{9}-THC- carboxylic acid ($CDCl_3$)

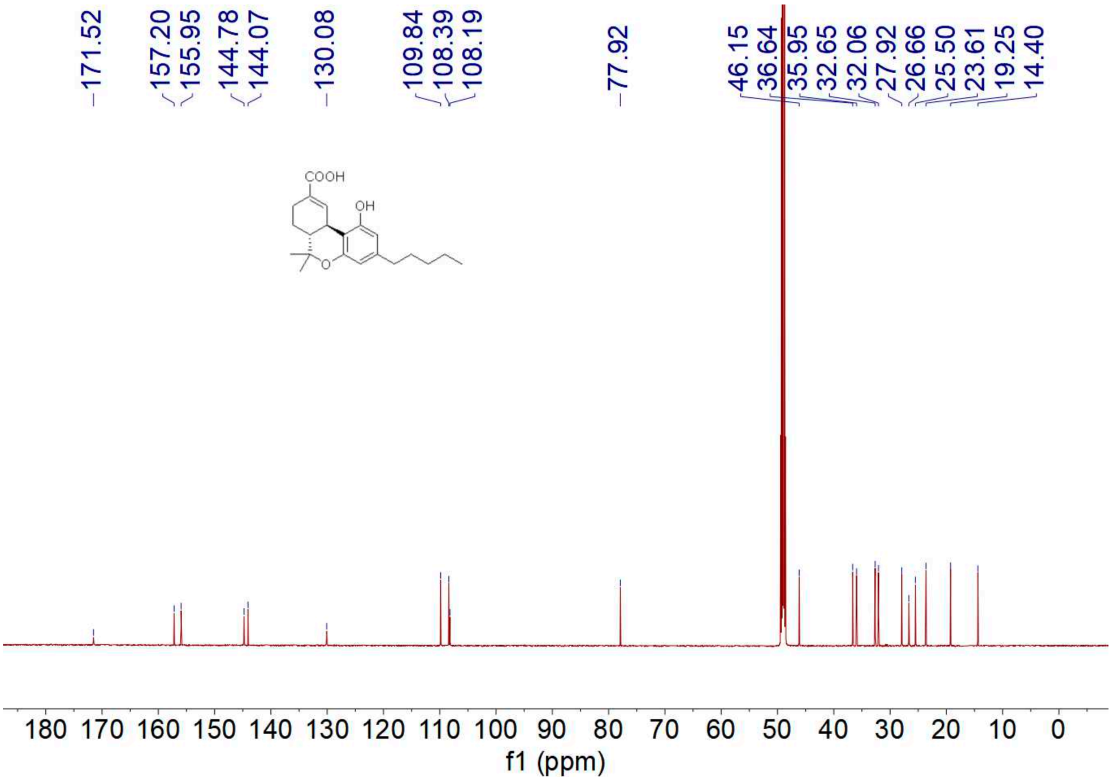

Figure S31 ^{13}C-NMR of compound Δ^{9}-THC- carboxylic acid ($CDCl_3$)
